# Upregulation of non-canonical and canonical inflammasome genes associates with pathological features in Krabbe disease and related disorders

**DOI:** 10.1093/hmg/ddac299

**Published:** 2022-12-15

**Authors:** María B Cachón-González, Chao Zhao, Robin J Franklin, Timothy M Cox

**Affiliations:** Department of Medicine, University of Cambridge, Level 5, PO Box 157, Cambridge CB2 0QQ, UK; Wellcome Trust-Medical Research Council Cambridge Stem Cell Institute, University of Cambridge, Cambridge CB2 0AW, UK; Department of Clinical Neuro sciences, University of Cambridge, Cambridge CB2 2PY, UK; Wellcome Trust-Medical Research Council Cambridge Stem Cell Institute, University of Cambridge, Cambridge CB2 0AW, UK; Department of Clinical Neuro sciences, University of Cambridge, Cambridge CB2 2PY, UK; Department of Medicine, University of Cambridge, Level 5, PO Box 157, Cambridge CB2 0QQ, UK

## Abstract

Infantile Krabbe disease is a rapidly progressive and fatal disorder of myelin, caused by inherited deficiency of the lysosomal enzyme β-galactocerebrosidase. Affected children lose their motor skills and other faculties; uncontrolled seizures are a frequent terminal event. Overexpression of the sphingolipid metabolite psychosine is a pathogenic factor, but does not fully account for the pleiotropic manifestations and there is a clear need to investigate additional pathological mechanisms. We examined innate immunity, caspase-11 and associated inflammatory pathways in twitcher mice, an authentic model of Krabbe disease. Combined use of molecular tools, RNAscope *in situ* hybridization and immunohistochemical staining established that the expression of pro-inflammatory non-canonical caspase-11, canonical caspase-1, gasdermin D and cognate genes is induced in nervous tissue. Early onset and progressive upregulation of these genes accompany demyelination and gliosis and although the molecules are scant in healthy tissue, abundance of the respective translation products is greatly increased in diseased animals. Caspase-11 is found in reactive microglia/macrophages as well as astrocytes but caspase-1 and gasdermin D are restricted to reactive microglia/macrophages. The inflammasome signature is not unique to Krabbe disease; to varying degrees, this signature is also prominent in other lysosomal diseases, Sandhoff and Niemann-Pick Type-C1, and the lysolecithin toxin model of focal demyelination. Given the potent inflammatory response here identified in Krabbe disease and the other neurodegenerative disorders studied, a broad induction of inflammasomes is likely to be a dominant factor in the pathogenesis, and thus represents a platform for therapeutic exploration.

## Graphical abstract

**Figure:**
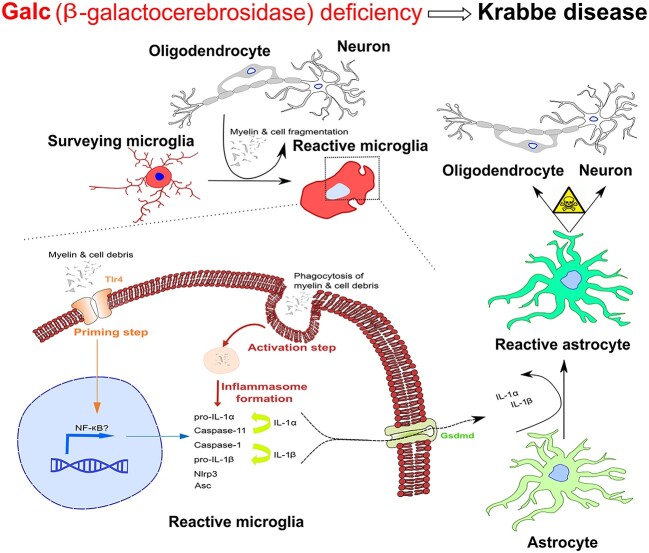

## Introduction

Krabbe disease (OMIM 245200), also known as globoid cell leukodystrophy (1), is an inborn error of metabolism caused by deficiency of the lysosomal enzyme β-galactocerebrosidase (GALC) (EC 3.2.1.46) (2), essential for the hydrolysis of the sphingolipid β-galactosylceramide (GalCer).

Myelin, the insulating sheath of nerve fibres, produced by oligodendrocytes and Schwann cells, is highly enriched in GalCer (3). A consequence of GALC deficiency is that the turnover of myelin is compromised, which ultimately leads to its degeneration and loss of myelin-producing cells (4,5). Many lysosomal diseases are characterized by the cellular accumulation of the target compound of the relevant defective enzyme, but in Krabbe disease, GalCer does not accumulate in myelin-producing cells. Rather, the cognate cytotoxic lipid metabolite galactosylsphingosine (psychosine), itself a substrate of GALC, increases progressively with disease severity (6,7). Psychosine is generated catabolically through deacylation of GalCer by acid ceramidase (N-acylsphingosine deacylase, EC 3.5.1.23) in the lysosome, partly explaining the lack of GalCer increases in diseased tissue (8).

The incidence of Krabbe disease is estimated at 1 in 100 000–250 000 births (9), and on the basis of the age of onset, infantile, juvenile and adult forms are recognized. In the most frequent and acute form, the infant appears normal at birth, but the disease declares itself within a few months: characteristic manifestations include extreme irritability, feeding difficulties, recurrent fevers, psychomotor regression and generalized seizures. Most of these children succumb before the age of 2 years (10). Juvenile and adult cases develop neurodegenerative signs but with later onset and an attenuated course (11). Hematopoietic stem cell transplantation, although not curative, is the only effective treatment for Krabbe disease, and long-term clinical benefit is restricted to those patients who undergo this procedure before symptomatic disease becomes apparent (12).

GALC is expressed by different cell-types in neural tissue (13), and infiltrating macrophages respond to demyelination by increasing GALC production, an indication of regulated expression (14). After a demyelinating event, owing to its inhibitory effect on remyelination and strong pro-inflammatory effects, myelin debris must be removed (15). Microglia/macrophages principally undertake this clearance; for receptor-mediated phagocytic removal, myelin binds to surface receptors either directly or after opsonization (16), but clearance of degenerated myelin cannot proceed effectively in Krabbe disease because of GALC deficiency in these cells.

Danger-associated molecular patterns are sensed by cytosolic innate immune receptors which, together with the adaptor molecule ASC (apoptotic speck-containing protein with a card) and pro-caspase-1 assemble into a canonical inflammasome complex ensuing pro-caspase-1 activation (17). Mature caspase-1 cleaves and activates many substrates, including interleukin-1β (IL-1β); a major mediator of pathogenesis that induces local inflammation, fever and interferon responses (18,19).

The more recently discovered non-canonical inflammasomes, consisting of mouse caspase-11 and its human orthologues caspase-4 and -5, are best known as endogenous receptors for endotoxin lipopolysaccharide (LPS), derived from the outer membrane of Gram-negative bacteria. LPS through its lipid A tail moiety binds caspase-11, and leads to its oligomerization and proximity-induced activation by auto-proteolysis (20–23). In common with canonical inflammasomes, non-canonical inflammasomes can cause cytokine release and pyroptosis mediated by gasdermin D (GSDMD). The C-terminal domain of the full size GSDMD auto-inhibits its function, but cleavage by either caspase-1 or -11 releases this inhibition. The GSDMD-N-containing fragment can travel to the plasma membrane to form membrane-permeable pores. Pore formation can result in loss of ionic gradient, cell membrane rupture and extravasation of cellular contents (24–26).

Autophagosomes and lysosomes are important mediators of innate immunity (27–29) and inborn errors that undermine the function of these organelles together with unhydrolyzed compounds as a result of an impaired autophagosomal/lysosomal system likely influence immune responses. An idea supported by the recent discovery that the canonical inflammasome Nlrp3 (nucleotide-binding leucine-rich repeat receptor family member pyrin-domain containing) is activated in diverse lysosomal disorders, Gaucher disease (30) and mucopolysaccharidosis type IIIA (MPSIIIA) (31), caused by defects in glucosylceramide and heparan sulphate degradation, respectively. On the basis of these and findings by others, we hypothesized that inflammasome activation and GSDMD-mediated pyroptosis might occur in Krabbe disease and contribute substantially to pathogenesis and neurological disability. Accordingly, we designed the current study with two specific goals: to elucidate the involvement of the inflammasome in Krabbe disease, and to understand whether there is an inflammasome signature characteristic of the disease. To assess the specificity of the inflammasome response in Krabbe disease, we used comparative analysis, against lysosomal disorders Sandhoff (SD) and Niemann–Pick Type C1 (NPC1) disease that preferentially target neurons, and a distinct model of focal demyelination caused by injection of the toxin lysolecithin (Lys) into the spinal cord of wild-type animals.

## Results

### Non-canonical and canonical inflammasome genes are upregulated in twitcher

**Figure 1:**
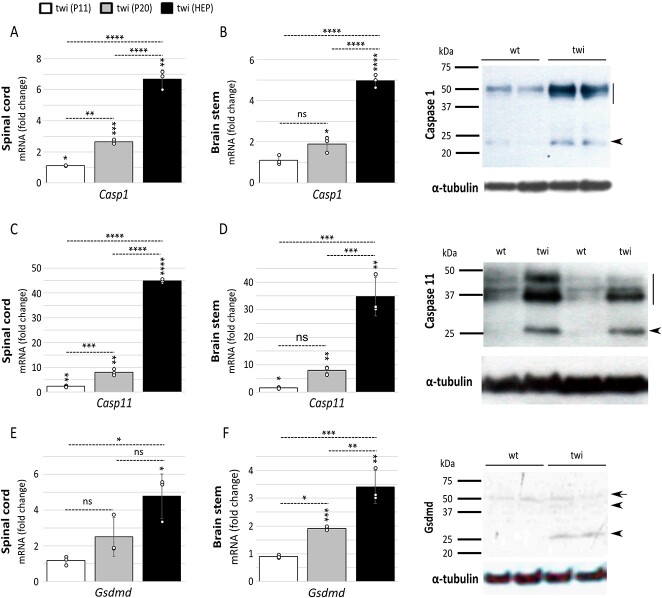
Pro-inflammatory caspases-1 and -11, and Gsdmd are induced and activated in twi. Gene expression was examined in spinal cord and brain stem by RT-qPCR at three stages of disease, 11 days (P11; pre-symptomatic), 20 days (P20; early symptomatic) and HEP (39–42 days) from twi and their wild-type (wt) counterparts (*n* = 3 distinct animals per group) performed in triplicate: (**A**-**B**) *Casp-1* (Caspase-1); (**C**-**D**) *Casp-11* (Caspase-11); and (**E**-**F**) *Gsdmd* (Gasdermin D). Data are expressed relative to wild-type animals as mean ± STDEV and tested by unpaired Student’s *t*-test. Symbols above error bars are twi values versus wild-type, and the absence of a symbol indicates the test did not reach significance. Comparisons between the different twi age groups were made using one-way ANOVA with Bonferroni’s multiple post hoc test. ^*^P ≤ 0.05; ^**^P ≤ 0.01; ^***^P ≤ 0.001; ^****^P ≤ 0.0001; ns, not significant. Protein tissue extracts taken from the brain stems of twi at the HEP and their wild-type counterparts were loaded into 12% PAGE gels, with each lane representing a different animal, and immunoblotted for: (B) caspase-1; (D) caspase-11; and (F) Gsdmd. Side bars and arrows denote un-cleaved protein species, and arrowheads cleaved forms. α-tubulin was used as control for protein loading.

We examined expression of inflammasome components in the nervous system of twitcher (twi) mice (*Galc^twi-2J/twi-2J^*). The twi-2J opal mutation causes a nonsense change at codon 339 (TGG > TGA) in *Galc* and totally abrogates the function of the enzyme (32). Twi recapitulates biochemical and neuropathological features of acute infantile Krabbe disease (4,33). Real-time (RT) quantitative PCR (RT-qPCR) of total RNA was used to define transcriptional expression at three key stages of disease progression: (1) 11 days [postnatal day 11 (P11); pre-symptomatic], (2) 20 days [postnatal day 20 (P20); early symptomatic] and (3) humane endpoint (HEP: 39–42 days). The spinal cord and brain stem were selected as paradigmatic sites of neurodegeneration and inflammation. These structures myelinate early in development and undergo early demyelination (4). We first studied the transcriptional expression profile of key inflammasome components, pro-inflammatory caspase-1 and -11, which we found significantly upregulated in both structures with increasing age, and therefore with disease progression (Fig. 1A–D). The high expression of caspase-11 in particular prompted further investigations. This is because caspase-11: (1) is not constitutively expressed by most cells due to the stringent regulation of its induction (20); (2) requires a priming step for its upregulation, which occurs following pathogen and cytokine receptor stimulation; and (3) its role in sterile neurodegeneration has hardly been explored (34).

Caspase-1 and -11 are synthesized as zymogens and require auto-proteolysis for their activation. Caspase-1 is translated as a ~ 45 kDa pro-enzyme (35), but a number of isoforms have been identified resulting in products of between 30 and 48 kDa (36). A ~ 20 kDa catalytically active and 10 kDa subunits are produced after auto-proteolysis (35). Increased protein expression of caspase-1 in twi was established by immunoblotting; pro-caspase-1 and mature p20 species are both elevated compared with wild-type controls (Fig. 1B).

Initial analysis of caspase-11 predicted a molecular mass of 42 kDa, but after LPS stimulation, unprocessed 38–43 kDa spliced variants and cleaved active forms, 26–30 kDa and 10 kDa, were identified (20). In agreement with findings by Wang *et al*. (20), we also observed small amounts of pro-caspase-11 and cleaved species in wild-type mice. In contrast, un-cleaved and cleaved forms of caspase-11 were markedly increased in twi, correlating with the RT-qPCR results (Fig. 1D).

Because overexpression and activation of caspase-1 and -11 can cleave Gsdmd and lead to pyroptosis (37), we studied the transcriptional profile of *Gsdmd* and found that its expression also increased with disease progression (Fig. 1E and F). Immunoblotting detected the 53 kDa full-size Gsdmd in mice of all genotypes, but the ~ 30 kDa cleaved active form was only observed in twi (Fig. 1F). An additional 43 kDa fragment was present in diseased animals, reported to result from cleavage by caspase-3 during apoptosis in other disorders (38).

**Figure 2:**
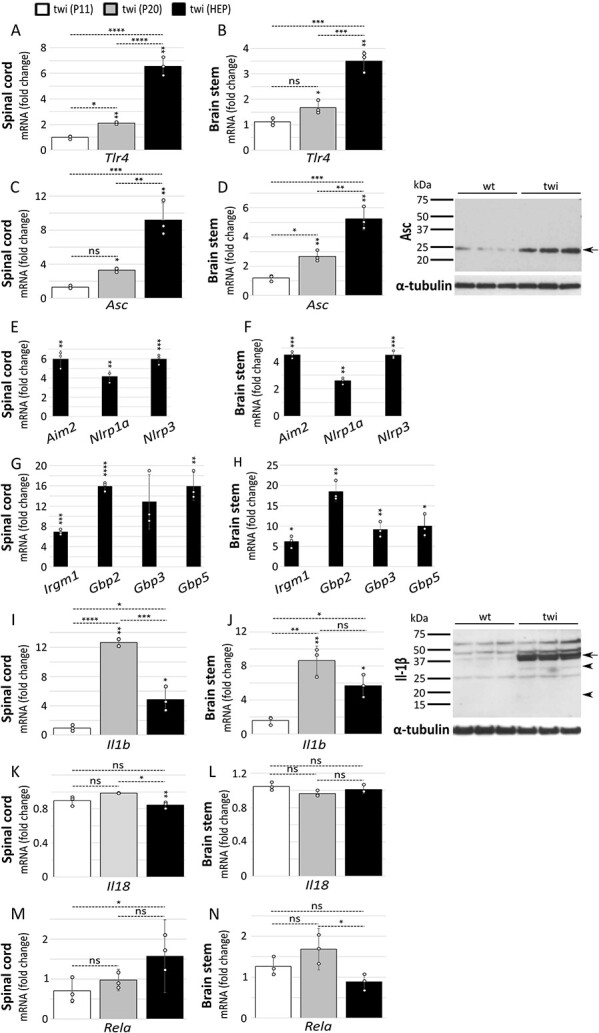
Expression of inflammasome-associated genes is upregulated in twi. Gene expression was examined in spinal cord and brain stem from twi and their wild-type (wt) counterparts (*n* = 3 distinct animals per group) by RT-qPCR performed in triplicate, at the humane end point (HEP) (39–42 days) only, or at three stages of disease, 11 days (P11; pre-symptomatic), 20 days (P20; early symptomatic) and HEP: (**A**-**B**) *Tlr4* (Toll-like receptor 4); (**C**-**D**) *Asc* (Apoptosis-associated speck-like protein containing a CARD); (**E**-**F**) *Aim2* (Absent in melanoma 2), *Nlrp1a* (Nucleotide-binding leucine-rich repeat receptor family member pyrin-domain containing 1a) and *Nlrp3* (Nucleotide-binding leucine-rich repeat receptor family member pyrin-domain containing 3); (**G**-**H**) *Irgm1* (Immunity-related GTPase family m1), *Gbp2* (Interferon-induced guanylate-binding protein 2), *Gbp3* (Interferon-induced guanylate-binding protein 3) and *Gbp5* (Interferon-induced guanylate-binding protein 5); (**I**-**J**) *Il1b* (Interleukin 1 beta); (**K**-**L**) *Il18* (Interleukin 18); and (**M**-**N**) *Rela* (Nuclear factor NF-kappa-B p65 subunit). Data are expressed relative to wild-type animals as mean ± STDEV and tested by unpaired Student’s *t*-test. Symbols above error bars are twi values versus wild-type. The absence of a symbol indicates the test did not reach significance. Comparisons between the twi age groups were made using one-way ANOVA with Bonferroni’s multiple post hoc test. ^*^P ≤ 0.05; ^**^P ≤ 0.01; ^***^P ≤ 0.001; ^****^P ≤ 0.0001; ns, not significant. Protein tissue extracts taken from the brain stems of twi at the HEP and their wild-type counterparts were loaded into 12% PAGE gels, with each lane representing a different animal, and immunoblotted for Asc (D) and Il-1β (J). Arrows in blots denote un-cleaved protein species, and arrowheads cleaved forms. α-tubulin was used as a control for protein loading.

We next investigated the transcriptional expression of inflammasome-associated genes Toll-like receptor 4 (*Tlr4*) and *Asc*. Tlr4 is known to be activated by sulfatide, an abundant sphingolipid of myelin (39), and Asc is required for canonical inflammasome activation. *Tlr4* and *Asc* are both progressively upregulated in twi (Fig. 2A–D). Moreover, immunoblotting against Asc showed increased amounts of protein in these mice (Fig. 2D). Because several canonical inflammasome sensors have been implicated in neurological disorders, including Aim2, Nlrp1a and Nlrp3 (40,41), we studied their transcriptional profile and found them similarly upregulated in twi (Fig. 2E and F).

It has emerged that LPS activation of caspase-11 depends on cellular factors with pivotal roles in host defence. IRGM (immune-related GTPases M clade) and GBPs (interferon-inducible GTPases guanylate-binding proteins) regulate autophagosome-lysosome fusion and lysis of vacuoles containing bacteria, respectively (42,43). Although the involvement of these molecules has not been examined in the context of sterile inflammation, we hypothesized that there might be unrecognized common pathways of non-canonical inflammasome activation whether induced by pathogen infection or sterile inflammation. Importantly, our results show the transcriptional expression of *Irgm1*, *Gbp2*, *Gbp3* and *Gbp5* is elevated in twi (Fig. 2G–H).

The 31 kDa IL-1β pro-form is not normally constitutively expressed in healthy states, but is potently induced by pro-inflammatory signals. Mature and functionally active 17 kDa IL-1β species can be generated by caspase-1 cleavage, and released at sites of infection/injury to regulate diverse physiological responses (35). The transcriptional expression of *Il1b*, but not the related family member *Il18*, was upregulated in twi (Fig. 2I–L). Interestingly, *Il1b* expression appears to be biphasic: of the three time points studied, we found that maximal transcription occurs at P20 and not at the HEP, although it remains significantly higher than at postnatal day 11 (P11). This finding was replicated in both structures studied here: spinal cord and brain stem, and thus unlikely to be an artefact. We currently do not have an obvious explanation for these results which appear counter-intuitive. Immunoblotting revealed a marked increase of the Il-1β pro-form and smaller amounts of the 17 kDa and 28 kDa cleaved species in twi (Fig. 2J). It is worth noting that a very low concentration of active Il-1β is sufficient to trigger downstream signalling. Furthermore, unlike in *in vitro* tissue culture experiments where cleaved Il-1β accumulates in the culture medium, the *in vivo* secreted Il-1β is unlikely to accumulate in large quantities as it is rapidly taken up by efficient receptors in surrounding cells.

The priming step of inflammasome activation mediated by pattern-recognition receptors often involves transcription factors such as NF-κB (nuclear factor kappa-light-chain-enhancer of activated B cells), to regulate the expression of components necessary for inflammasome formation (44). Examination of the transcriptional profile of *Rela*, which codes for the p65 subunit of NF-κB, did not revealed a clear significant difference between twi and its wild-type counterparts in either of the structures examined (Fig. 2M and N).

Taken together our results suggest an early onset and increased induction of non-canonical and canonical inflammasomes genes and associated molecules concomitant with disease severity and progression in the twi mouse.

### Inflammasomes and their associated genes are also upregulated in other neurodegenerative lysosomal storage diseases

To determine whether the inflammasome signature of Krabbe disease is specific to the pathophysiology of this disorder, we carried out similar investigations in authentic murine models of two additional lysosomal storage diseases (LSDs): SD and NPC1, which develop pathological features that preferentially target neurones.

Tay-Sachs and SD are caused by an impairment in the lysosomal enzymatic function of the alpha and beta subunits of β-N-acetylhexosaminidase, encoded by *HEXA* and *HEXB*, respectively. The two subunits dimerize to give rise to three isoforms: β-hexosaminidase A (HEX A), a heterodimer of alpha and beta subunits; β-hexosaminidase B (HEX B), a beta subunit homodimer; and β-hexosaminidase S (HEX S), an alpha subunit homodimer (45). Mutations in either *HEXA* or *HEXB* can cause fatal neurodegeneration with clinical courses that are, for the most part, indistinguishable. HEXA is absolutely required for the hydrolysis of GM2 ganglioside, a glycosphingolipid that accumulates predominantly in neurones when the isozyme is defective. The Sandhoff mouse was generated by targeted disruption of the *Hexb* gene (B6; 129S4-*Hexb^tm1Rlp^*/J) (46), and closely recapitulates the human condition clinically and biochemically. Its natural lifespan is around 4 months.

NPC disease is also a progressive neurodegenerative LSD, which principally impacts the brain (47). It is caused by mutations in either of two *NPC* genes (48). NPC1 is a lysosomal transmembrane protein and NPC2 a soluble protein, and are jointly responsible for the egress and recycling of lipoprotein-derived cholesterol from late endosomes/lysosomes toward other cellular compartments (49). Disease-causing mutations in either of the *NPC* genes lead to lysosomal accumulation of various lipids, including unesterified cholesterol, glycosphingolipids, sphingomyelin and sphingosine (50). A murine model of NPC1 (*Npc1^−/−^*, BALB/*cNctr-Npc1^m1N^/J*) carries a spontaneous loss of function mutation in the *Npc1* gene and develops classical NPC neurological disease, including the loss of Purkinje cells and an almost disease-free spinal cord. Death occurs at age 10–12 weeks (51,52).

**Figure 3:**
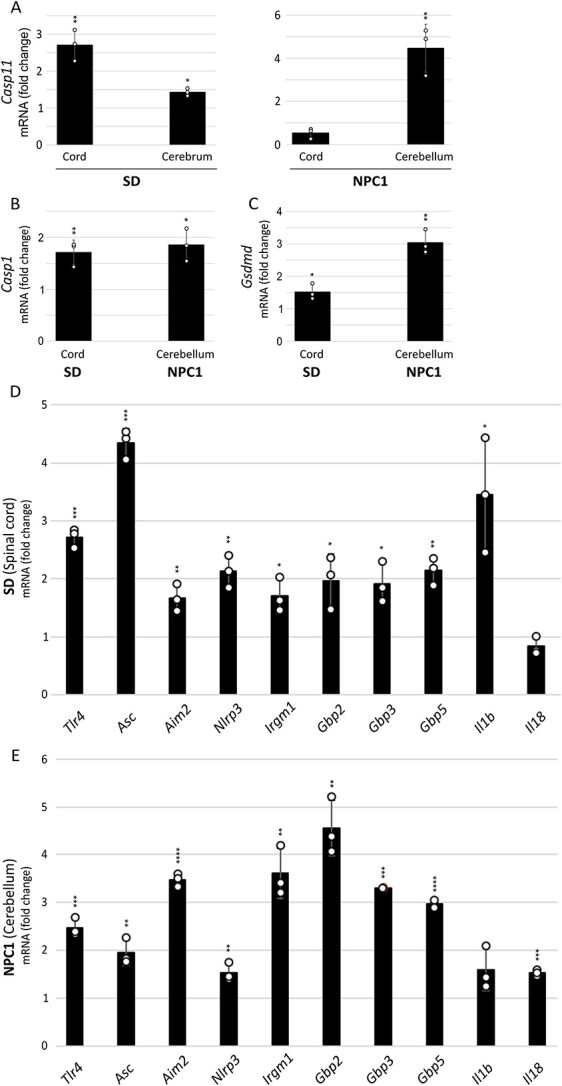
Expression of pro-inflammatory caspases and associated genes is also upregulated in other lysosomal storage diseases. Gene expression was examined by RT-qPCR at the humane end point (HEP) in mutant animals and their wild-type (wt) counterparts (*n* = 3 distinct animals per group) in triplicate. (**A**) *Casp-11* (Caspase-11), spinal cord and cerebrum from SD and spinal cord and cerebellum from NPC1 murine models; (**B**) *Casp-1* (Caspase-1) and (**C**) *Gsdmd* (Gasdermin D), spinal cord from SD and cerebellum from NPC1 mice; (**D**) SD spinal cord and (**E**) NPC1 cerebellum for: *Tlr4* (Toll-like receptor 4), *Asc* (Apoptosis-associated speck-like protein containing a CARD), *Aim2* (Absent in melanoma 2), *Nlrp3* (Nucleotide-binding leucine-rich repeat receptor family member pyrin-domain containing 3), *Irgm1* (Immunity-related GTPase family m1), *Gbp2* (Interferon-induced guanylate-binding protein 2), *Gbp3* (Interferon-induced guanylate-binding protein 3), *Gbp5* (Interferon-induced guanylate-binding protein 5); *Il1b* (Interleukin 1 beta) and Il18 (Interleukin 18). Data are expressed relative to wild-type animals as mean ± STDEV and tested by unpaired Student’s *t*-test. Symbols above error bars are mutant values versus wild-type. The absence of a symbol indicates the test did not reach significance. ^*^P ≤ 0.05; ^**^P ≤ 0.01; ^***^P ≤ 0.001 and ^****^P ≤ 0.0001.

We first studied the transcriptional expression of *Casp11* in the spinal cord and cerebrum or cerebellum of SD and NPC1, respectively, at their HEP by RT-qPCR. SD mice showed significant upregulation in both neural structures, with the spinal cord having more abundant expression (Fig. 3A). Consistent with a disease-free structure, transcript levels of *Casp11* in NPC1 mice were not increased in the spinal cord, but were markedly augmented in the cerebellum (Fig. 3A). On the basis of these findings, we then investigated the expression of other inflammasomes and associated genes in the spinal cord of SD and cerebellum of NPC1 mice. Transcript expression of most of the studied genes was upregulated in both murine models of disease (Fig. 3D and E).

Given the well-documented pathogenic role played by excessive inflammasome activation in a variety of neurodegenerative conditions, we conclude that our findings strongly suggest inflammasome activation as a likely pathogenic factor common to all the LSDs studied here: Krabbe disease, SD and NPC1. Nevertheless, the degree of upregulation is strikingly different, being most pronounced in the Krabbe disease model, which we attribute to the highly inflammatory cardinal features of the disease: death of oligodendrocytes/Schwann cells and severe demyelination.

### The upregulation of inflammasome gene expression correlates with the level of tissue degeneration and gliosis

Having established that canonical and non-canonical inflammasome expression in the twi mouse is characterized by an early onset and intensifies with age and disease progression, we examined possible associations with classical features of disease: demyelination and gliosis.

**Figure 4:**
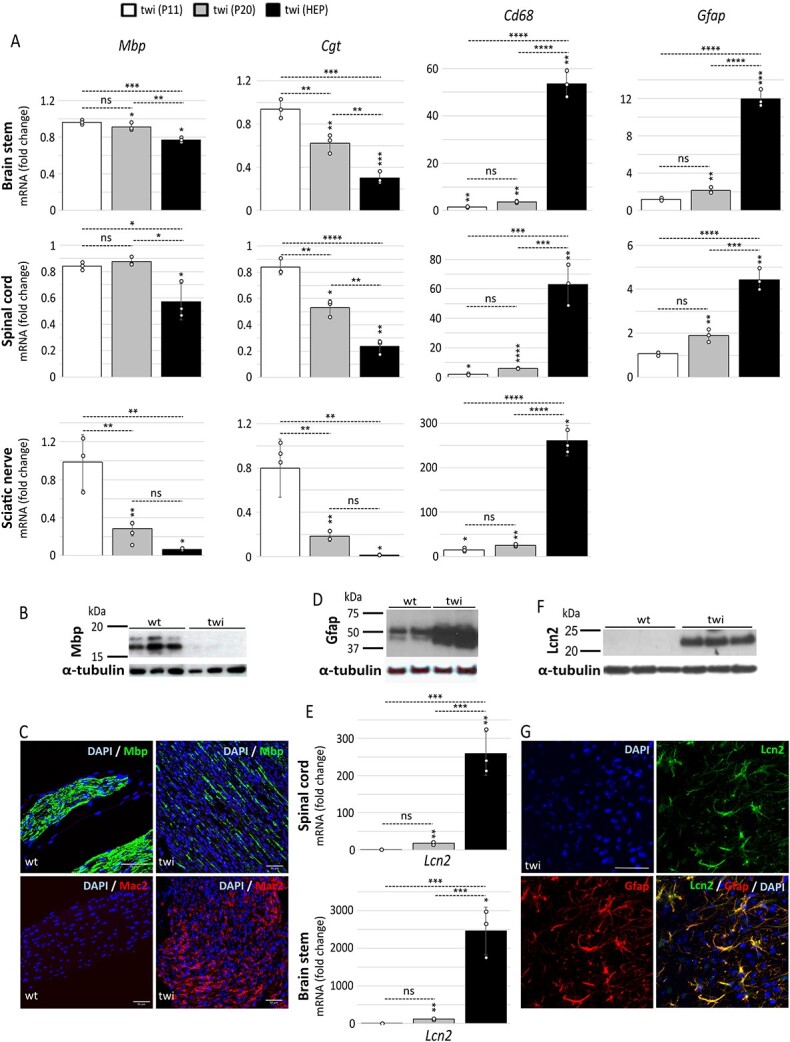
The spatiotemporal pattern of upregulated inflammasome gene expression mirrors demyelination and reactive gliosis in twi. (**A**) The expression of genes essential for myelination, *Mbp* (Myelin basic protein) and *Cgt* (UDPgalactose:ceramide galactosyltransferase), or indicative of reactive microgliosis, *Cd68* (Cluster of differentiation 68), and astrogliosis, *Gfap* (Glial fibrillary acidic protein) and *Lcn2* (Lipocalin 2) in **E**, were examined by RT-qPCR at three stages of disease: 11 days (P11; pre-symptomatic), 20 days (P20; early symptomatic) and HEP (39–42 days) in brain stem, spinal cord, and sciatic nerve from twi and their wild-type (wt) counterparts (*n* = 3 distinct animals per group) and performed in triplicate. Data are expressed relative to wild-type animals as mean ± STDEV and tested by unpaired Student’s *t*-test. Symbols above error bars are twi values versus wild-type. The absence of a symbol indicates the test did not reach significance. Comparisons between the twi age groups were made using one-way ANOVA with Bonferroni’s multiple post hoc test. ^*^P ≤ 0.05; ^**^P ≤ 0.01; ^***^P ≤ 0.001; ^****^P ≤ 0.0001; ns, not significant. Protein tissue extracts of sciatic nerve (**B**) or brain stem (**D**, **F**) from twi at the HEP and their wild-type counterparts were loaded into 15% (B) and 12% (D, F) PAGE gels, with each lane representing a different animal, and immunoblotted for: (B) Mbp, (D) Gfap and (F) Lcn2. α-tubulin was used as a control for protein loading. Representative sections of sciatic nerve from twi at the HEP and their wild-type counterparts were immunohistologically stained for Mbp and the reactive microglia/macrophages marker Mac2 (galectin 3) (**C**). Brain stem was co-stained with Gfap and Lcn2 (**G**). Nuclear stain is DAPI. Scale bar: 50 μm.

*Mbp* and *Cgt* code for myelin basic protein and UDP-galactose:ceramide galactosyl-transferase (EC 2.4.1.45), respectively. Mbp is an abundant structural protein of compact myelin and Cgt is the enzyme responsible for the biosynthesis of galactocerebrosides and sulfatides, thus these are proteins essential for normal myelination. Defects in myelin turnover and gliosis were examined by RT-qPCR in brain stem, spinal cord and sciatic nerve at the same three stages of disease as those assessed for inflammasome induction (P11, P20 and HEP), and in the same individual animals. There was a statistically significant decrease in *Mbp* transcripts in the spinal cord of twi compared with its wild-type counterparts at the HEP, but not at earlier time points. However, in brain stem and sciatic nerve, a decrease in transcript abundance was already apparent at P20, with the greatest reduction detected in sciatic nerve (Fig. 4A). *Cgt* expression was also lower at P20 and the HEP for all structures analysed, but not at P11 (Fig. 4A). The analysis showed that at the HEP, transcript levels for proteins enriched in normal myelin are depleted in twi, most prominently in sciatic nerve, which we also studied by immunoblotting and immunohistochemical staining against the protein Mbp (Fig. 4B and C).

Modelling studies in mice suggest that microglia represents the first line of defence against noxious stimulus; fast recruitment to sites of damage accompanied by cellular changes, from a surveying to a reactive phenotype, are common features. Secondary to the emergence of reactive microglia is the activation of astrocytes, which secrete inflammatory mediators that signal to microglia and recruit other cells, including monocyte-derived macrophages (53). We assessed the level of gliosis in the same structures, stages of disease and individual animal samples as in the above studies, by examining *Cd68* (Cluster of differentiation 68) and *Gfap* (Glial fibrillary acidic protein) transcript expression. Cd68 is a transmembrane glycoprotein expressed by monocytes and tissue macrophages, and its overexpression is indicative of phagocytic activity. Gfap is an essential type III intermediate filament protein and its abundance is enhanced in reactive astrocytes. *Cd68* transcript expression was significantly elevated in twi compared with its wild-type counterparts in all three structures and starting as early as at P11, the earliest time point examined, but it was found massively increased at the HEP: tens of folds greater for brain stem and spinal cord and hundreds of folds for sciatic nerve compared with P11 and P20 (Fig. 4A). Induction of *Gfap* expression followed a similar trend in brain and spinal cord (Fig. 4A). Immunohistochemical staining for the microglia/macrophage marker Mac2 (Fig. 4C) and Gfap (Fig. 4G), as well as immunoblotting against Gfap (Fig. 4D) support the RT-qPCR findings.

Because a large array of stimuli can lead to astrocyte reactivity with similar degrees of *Gfap* upregulation, while at the same time causing vastly different changes in transcriptome profile and cell function (54), we investigated the expression of *Lcn2*. Lipocalin-2 (Lcn2) is an acute phase protein of reactive astrocytes (55) which has been shown to be upregulated by Tlr4 (56) and is a potent mediator of neurotoxicity (57). *Lcn2* transcript expression was significantly higher, compared with wild-type animals, in the spinal cord and brain stem of twi; starting at P20 and increasing greatly with age (Fig. 4E). Moreover, *Lcn2* increased transcription levels directly correlated with protein quantities assessed by immunoblotting and immunohistochemical staining (Fig. 4F–G).

Statistical comparisons of gene expression using one-way analysis of variance (ANOVA) demonstrated overall significant differences between the age groups of twi. This led us to speculate that as myelin and myelin producing cells degenerate over time and cannot be effectively cleared by phagocytic cells—microglia/macrophages and to a lesser extent astrocytes—because of an intrinsic defect in GALC function, the severity of the reactive response mirrors tissue degeneration.

Given the primary biochemical defect, physiopathology and clinical course differ between LSDs, we reasoned the reactive phenotype of glia might be disease-specific, and be reflected in their transcriptomes. We compared the expression profile of a number of genes on the basis of the dataset from Liddelow *et al*. (58). We studied the spinal cord in twi and SD, and cerebellum in NPC1. Overall, the most prominent reactive phenotype occurred in twi, followed closely by NPC1, with SD mice having the least severe phenotype (Supplementary Material, Figure S1A–C).

The degree of expression of inflammasome-related genes correlates with phenotype severity and extent of reactive microglia/macrophages and astrocytes in the different murine models of disease studied here, thus suggesting an intimate relationship between cellular reactivity and inflammasome activation in these diseases; a phenomenon shared with other neurodegenerative conditions.

### Caspase-1 and *Gsdmd* are expressed principally in reactive microglia/macrophages and caspase-11 in microglia/macrophages as well as astrocytes

**Figure 5:**
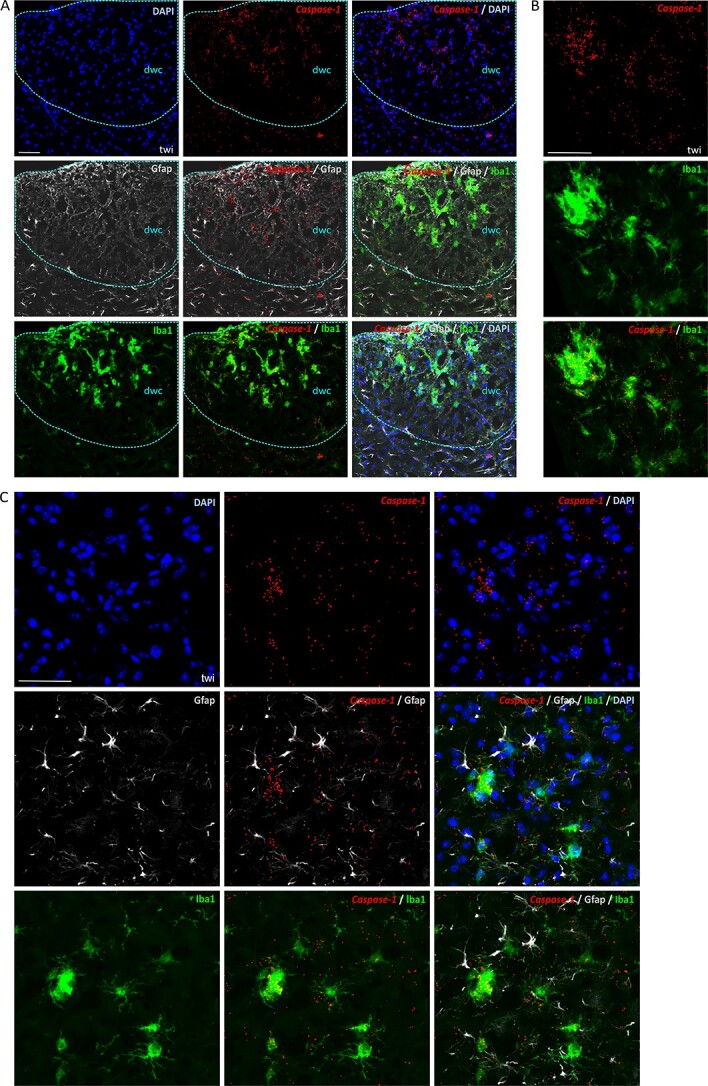
Canonical caspase-1 localizes to reactive microglia/macrophages in twi. Representative sections of spinal cord (**A**, **B**), and brain stem (**C**) from twi at the humane end point, co-stained by ISH for caspase-1 mRNA and immunohistochemically for astrocytes with Gfap (Glial fibrillary acidic protein) and microglia/macrophages with Iba1(Ionized calcium binding adaptor molecule 1). Nuclear stain is DAPI. Spinal cord dorsal white column (dwc) is outlined with blue dashes. Scale bars: 50 μm.

We examined tissue distribution and cell-type specific expression of *Casp1, Casp11* and *Gsdmd* in brain, spinal cord and nerves from twi at the HEP by *in situ* hybridization (ISH) using messenger RNA (Ribonucleic acid) (mRNA) mouse-specific RNAscope probes because the antibodies used for immunoblotting proved unsuccessful for immnunohistochemical (IHC) staining. We combined ISH with IHC staining for cell-specific markers. The microglia/macrophages of wild-type mice are immunoreactive for Iba1 (ionized calcium-binding adapter molecule 1) and display the characteristic surveying phenotype. They are evenly distributed throughout the tissue, and present with a small soma and highly branched processes (Supplementary Material, Figure S2A). In these animals, *Casp1* is expressed at low levels (Supplementary Material, Figure S2A). However, Iba1 immunoreactivity in twi is most intense in areas of white matter; for example, in the dorsal column of the spinal cord (Fig. 5A and B), brain stem (Fig. 5C) and sciatic nerve (Supplementary Material, Figure S2B); regions presumed to contain degenerated myelin/cell debris. The microglia/macrophages in twi tend to cluster, have an amoeboid morphology and thicken processes, and *Casp1* signal is prominent in the nucleus, cytoplasm and cell processes, but generally absent in cells with a well ramified morphology (Fig. 5A-C). Co-staining of *Casp1* with Gfap (Fig. 5A and C), Olig2 (oligodendrocyte transcription factor 2) (Supplementary Material, Figure S2C) or SMI32 (Supplementary Material, Figure S2C) shows little or no co-localization. SMI32 is an antibody specific for hypophosphorylated neurofilament H protein, which normally resides in neuronal bodies and processes. We explored *Casp1* and SMI32 co-reactivity because we recently described a neuronal cell population in twi positive for SMI32 which also accumulates abnormal aggregates of p62 (encoded by *Sqstm1*), a protein with important roles in ubiquitin/proteosomal and autophagosomal/lysosomal functions (59).

Expression of *Casp1* in hindbrain and spinal cord of SD mice is generally low, but Iba1-positive cells with a reactive morphology stain strongly (Supplementary Material, Figure S3A). Co-labelling with Gfap is not apparent (Supplementary Material, Figure S3A and B), and neurones, even when laden with large quantities of unhydrolyzed compounds, show no *Casp1* staining either (data not shown). We noted *Casp1* signal localizing to the Purkinje cell layer and spreading into the molecular cell layer of the cerebellum (Supplementary Material, Figure S3B-C), but we are yet to establish whether these are Purkinje cells or Bergmann glia as they generally align closely to the soma of Purkinje cells and their ascending processes. In NPC1 mice, *Casp1* in the cerebellum localizes principally to the white matter, but does not appear to co-localize with Gfap (Supplementary Material, Figure S3D). Although we presume increased expression of *Casp1* occurs in reactive microglia/macrophages in mutant NPC1, we could not establish this fact; staining with Iba1, Cd68 or Mac2 proved unsuccessful in the ISH/IHC combined application when using fresh-frozen brain sections, the only available material we had from these animals. We should point out, however, that because reactive microglia/macrophages and astrocytes are normally in close proximity, we cannot exclude the possibility that small quantities of *Casp1* might also be present in astrocytes.

To examine whether the strong inflammasome gene expression observed during the course of disease progression in the twi was shared with a different model of demyelination, we studied *Casp1* expression in wild-type mice that had been injected with lysolecithins in the white matter of the ventral spinal cord and killed 5 days post-injection. Lysolecithins (lysophosphatidylcholines) are metabolites of phospholipids, normal components of cell membranes. Nevertheless, localized injections of lysolecithins in the mouse spinal cord cause demyelination, selectively killing oligodendrocytes in the injected and surrounding area (60). Analysis of tissue sections proximal to the injection site shows high *Casp1* expression co-localizing with reactive Iba1-positive cells, but no obvious *Casp1* staining was found to co-localize with Gfap, nor with Olig2-labelled cells (Supplementary Material, Figure S3E-F).

**Figure 6:**
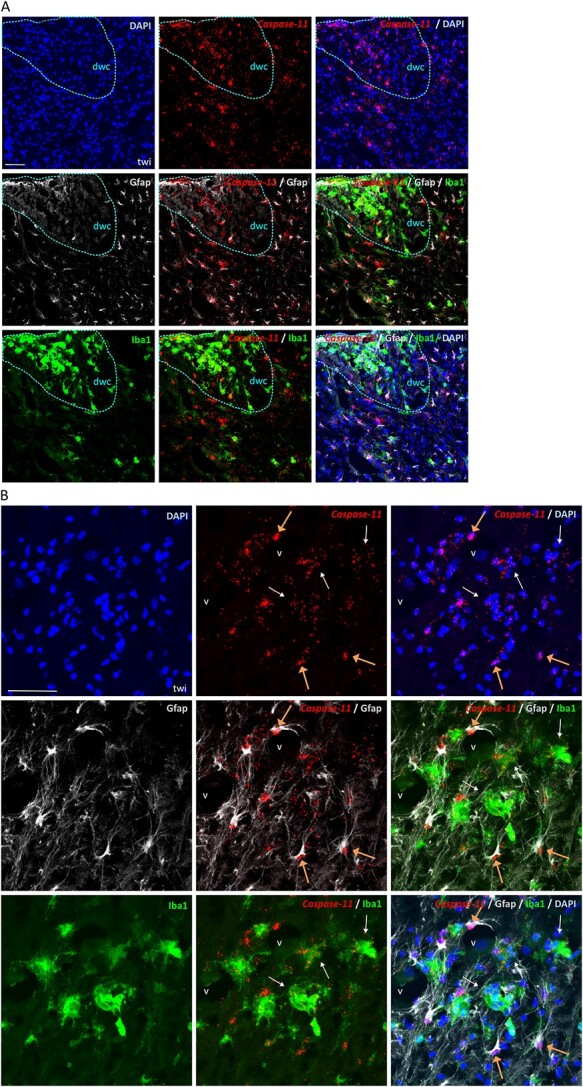
Non-canonical caspase-11 localizes to both reactive microglia/macrophages and astrocytes in twi. Representative sections of spinal cord (**A**) and brain stem (**B**) from twi at the HEP, co-stained by ISH with caspase-11 mRNA and immunohistochemically for astrocytes with Gfap (Glial fibrillary acidic protein), and microglia/macrophages with Iba1 (Ionized calcium binding adaptor molecule 1). Nuclear stain is DAPI. Dorsal white column of spinal cord (dwc) is highlighted with blue dashes; V (blood vessel). White and orange arrows point to microglia/macrophages and astrocytes, respectively. Note the different cellular distribution of *Casp-11* signal between microglia/macrophages and astrocytes. Scale bars: 50 μm.

Expression of *Casp11* is extremely low in samples from wild-type mice (Supplementary Material, Figure S4A), but is abundant in twi in areas presumed to contain degenerated myelin/cell debris, such as white matter of the dorsal column of the spinal cord (Fig. 6A), brain stem (Fig. 6B) and sciatic nerve (Supplementary Material, Figure S4B). These results mirror our earlier observations of *Casp1*-induced expression. However, unlike that of *Casp1* which is mostly limited to reactive Iba1-positive microglia/macrophages, *Casp11* is also detected in Gfap-stained astrocytes, characterized by thicken processes that often surround, or are in close proximity to, reactive microglia/macrophages (Fig. 6A and B). Of note, *Casp11* signal has a dispersed distribution in reactive microglia/macrophages, but localizes tightly to the nucleus in astrocytes. In contrast, Oligo2- and NeuN-stained cells are not positive for *Casp11* in twi (Supplementary Material, Figure S4B-C). Similar patterns of *Casp11* expression are prominent in reactive microglia/macrophages and astrocytes in the brains and spinal cords of SD mice (Supplementary Material, Figure S5A-B), and in wild-type mice injected with Lys (Supplementary Material, Figure S5D-E). In NPC1 mutant mice, *Casp11* is particularly conspicuous in the white matter of the cerebellum in astrocytes and other cell types, presumably microglia/macrophage (Supplementary Material, Figure S5C). No *Casp11* was identified in the Purkinje cell layer or neurones in SD or NPC1 mice (data not shown), nor in cells stained with Olig2 in SD, NPC1 or Lys-injected animals (Supplementary Material, Figure S5F).

**Figure 7:**
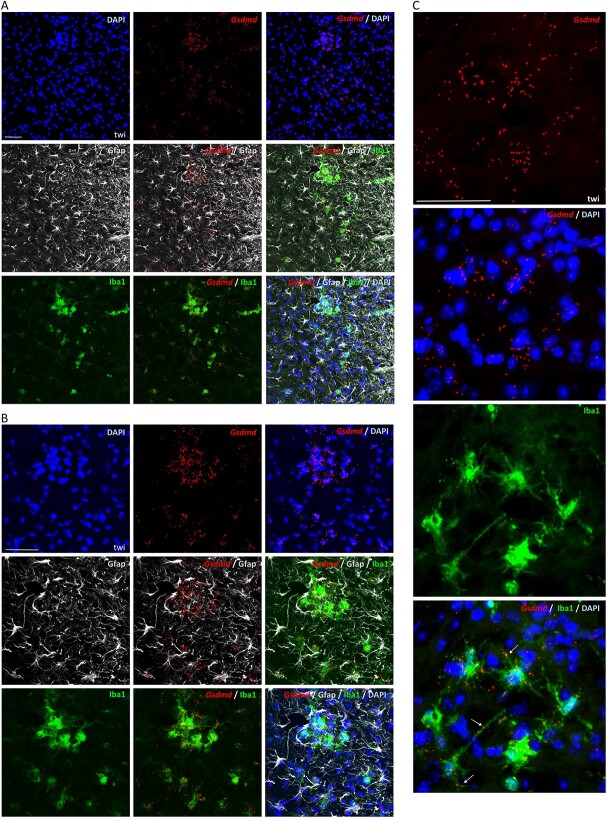
*Gsdmd* localizes principally to reactive microglia/macrophages in twi. (**A**-**C**) Representative sections of brain stem from twi at the HEP, co-stained by ISH with *Gsdmd* mRNA and immunohistochemically for astrocytes with Gfap (Glial fibrillary acidic protein) and microglia/macrophages with Iba1 (Ionized calcium binding adaptor molecule 1). Nuclear stain is DAPI. White arrows point to processes in microglia/macrophages containing *Gsdmd* signal in C. Scale bars: 50 μm.

Expression of *Gsdmd* is also low in wild-type mice (Supplementary Material, Figure S6A), but is highly upregulated in brain stem, spinal cord and nerves of twi, with a distribution similar to *Casp1* and *Casp11*, in areas presumed to be undergoing demyelination (Fig. 7A–C and Supplementary Material, Figure S6B-D). *Gsdmd* localizes principally to reactive Iba1-stained microglia/macrophage in nucleus, cytoplasm and cellular processes, and to a much lesser extent to astrocytes (Fig. 7A and B). No *Gsdmd* signal is observed in either Olig2 or NeuN-stained cells (Supplementary Material, Figure S6C-D). In SD and Lys-injected wild-type mice, *Gsdmd* is abundant in reactive microglia/macrophage (Supplementary Material, Figure S7A, C-D), and it was also abundant in mutant NPC1 animals (Supplementary Material, Figure S7B).

We conclude that upregulated expression of *Casp1* and *Gsdmd* occurs principally in reactive microglia/macrophages, and *Casp11* in both microglia/macrophages and astrocytes in diseased tissue of all models of disease studied here, with twi and the Lys toxin model of demyelination showing the strongest expression. The spatial distribution of *Casp1*, *Casp11* and *Gsdmd* overlaps and appears specific to regions suspected of undergoing degeneration.

### Myelin debris and associated sphingolipids do not prime the expression of caspase-11 in culture

To test whether myelin debris or associated sphingolipids, galactosylceramide and galactosylsphinosine (psychosine), are instrumental in priming the expression of caspase-11 in twi mice, we isolated astrocytes from the brains of neonatal mice and cultured them *in vitro*. Enriched primary astrocyte cultures isolated from individual animals, wild-type or mutant for *Galc*, were split and grown in medium containing one of the following compounds: (1) LPS (100 ng/ml); (2) myelin debris (5 μg/ml); (3) galactosylsphingosine (10 μM); (4) galactosylceramide (10 μM); and (5) culture medium (UT: untreated).

LPS, a known inducer of *Casp11*, was used as a control. LPS treatment of astrocytes, whether wild-type or mutant for *Galc* (twi), resulted in abundant expression of *Casp11*; detected by ISH using the same mRNA probe as that applied to tissue sections, and combined with IHC staining for Gfap. LPS induced the expression of *Casp11* in most Gfap-immunoreactive cells. In addition, other cell types not stained with Gfap also showed *Casp11* expression, which we presume to be microglia/macrophages, as the cultures were enriched but not pure for astrocytes. Treatment with myelin debris, galactosylsphingosine or galactosylceramide resulted in hardly any expression of *Casp11* (Supplementary Material, Figure S8). The principal difference between astrocyte cultures treated with galactosylsphingosine or galactosylceramide was that galactosylsphingosine triggered extensive cell death, which is in agreement with findings by others (61).

We conclude that neither myelin debris nor galactosylsphingosine or galactoceramide are able to induce the expression of caspase-11 on their own during the *in vitro* culture of primary astrocytes, whether wild-type or mutant for *Galc*. This suggests that different or additional stimuli are required for priming caspase-11 expression *in vitro*.

## Discussion

Krabbe disease, an inborn error of sphingolipid metabolism, is characterized by florid demyelination and neuroinflammation. The deficiency of GALC activity in this disorder leads to the overproduction of the neurotoxic lysosphingolipid galactosylsphingosine, which is derived by deacylation of galactosylceramide as a consequence of activation of acid ceramidase (N-acylsphingosine deacylase, ASAH1) in the pathological lysosome (8). Although this metabolite, commonly known as psychosine, contributes to the neuropathology of Krabbe disease (6,7,61), as demonstrated by interbreeding twi mice with a strain genetically engineered with Asah1 deficiency, or by administration of carmofur (an inhibitor of psychosine biosynthesis), these measures did not completely correct the neurological phenotype, nor did they fully restore survival in mutant animals (8). Although psychosine is clearly toxic, its accumulation cannot account for all the manifestations of this disease—a view supported by experimental data (62–67). Results from these and other studies suggest that the pathological features of the disease are the consequence of several cellular and molecular pathways, induced at least in part by psychosine and other, largely unrelated inflammatory events.

We speculated that exposure to myelin/cell debris, whether extra- or/and intracellularly, might engage innate sensors of damage-associated molecular patterns and drive inflammasome activation, thus contributing to pathogenesis. Expression of caspase-1 and caspase-11 increases progressively with age and disease severity in twi mice, and both pro-forms and cleaved species are conspicuous in nervous tissue, suggesting inflammasome activation.

Pro-inflammatory caspases cleave Gsdmd and can lead to pyroptosis and cytokine release. *Gsdmd* is upregulated in neural tissue in twi mice and parallels the expression pattern of pro-inflammatory caspases. The cleaved Gsdmd form is presumably able to form pores in the plasma membrane, but even if pores are formed, this might not inevitably provoke cell death. Exposure of phagocytes to sublytic amounts of inflammasome activation can cause pore formation and release of cytokines without committing the cell to die (68). Although canonical inflammasomes and Gsdmd have been implicated in other neurodegenerative conditions (69,70), the finding of caspase-11 induction, particularly strong in the Krabbe disease model was unexpected, as its activation is best known in response to bacterial infections. Only a small number of studies have examined caspase-11 induction in brain under sterile conditions (71,72). Our data show that remarkably, pathological events in Krabbe disease induce the expression of caspase-11 and associated molecules *Gbps* and *Irgm*, known as the most highly expressed and important genes in combating infection (43,73–75).

To stablish the unique nature, or otherwise, of the inflammasome signature in Krabbe disease, we performed comparative studies with mouse models of the lysosomal storage diseases Sandhoff and NPC1. The expression of inflammasome and associated genes is significantly upregulated in all three disorders, albeit showing different degrees of activation. This would suggest that exposure of innate immune cells to degenerated/dead oligodendrocytes/Schwann cells and myelin debris in Krabbe disease is, in all likelihood, the driver of the identified strong inflammasome response.

Because inflammasome activation is not limited to professional innate immune cells, we combined mRNA ISH with IHC staining to establish tissue and cellular distribution of key components. Expression of *Casp1*, *Casp11* and *Gsdmd* is extremely low in wild-type animals, and whereas *Casp1* and *Gsdmd* are mostly confined to reactive microglia/macrophages, *Casp11* is also highly induced in reactive astrocytes. However, the *Casp11* signal congregates almost exclusively in the nucleus of astrocytes, while appearing evenly distributed in microglia/macrophages. Whether the nucleus-retained RNA is unspliced or mature and what the biological significance might be is currently unknown, but genome-wide studies have reported that many mRNAs are retained in the nucleus at high levels (76,77), and can be released into the cytoplasm upon stimulation, suggesting a novel control mechanism of gene expression (78).

A study on the Experimental Autoimmune Encephalomyelitis murine model of demyelination demonstrated increased expression of inflammasome-associated genes, and importantly, many of the findings were corroborated in autopsied samples from patients with multiple sclerosis (70). Caspase-1 and Gsdmd were identified in cells immunoreactive for Iba1 and glutathione S-transferase pi, an oligodendrocyte marker. Although not discussed directly, the report showed *Casp11* upregulation in these mice, and attenuated expression following exposure to the inhibitor VX-765. Improvement in tissue injury and behaviour were attributed to caspase-1 inhibition. However, it can be argued that the beneficial outcome might be due, at least in part, to inhibition of caspase-11, because VX-765 also hinders the function of this proteinase (79).

Functionally active interleukin-1, IL-1α and IL-1β, are produced after inflammasome activation. Il-1α is specifically cleaved and activated by caspase-11 in macrophages (80), and we speculate also in microglia. These powerful apical cytokines bind and signal through type 1 IL-1 receptor (IL-1R), which can be blocked by the competitive action of the IL-1 receptor antagonist (IL-1Ra), inhibiting all signal transduction (81). The interleukin-1 system, mediated by microglia/macrophage, contributes to neurodegeneration and its inhibition improves disease outcome (82,83). Notably, within the context of a lysosomal disease, overexpression of IL-1Ra or abrogation of Il-1R1 in a mouse model of MPSIIIA halted the development of pathological hallmarks, including a striking reduction in reactive astrocytes (31), which is evidence of interleukin-1 being a driver of astrogliosis (84). Importantly, the salutary effects occurred without a reduction in the accumulation of storage substrates. The results from the MPSIIIA study are well aligned with those of Liddelow *et al*. on the activation of astrocytes by microglia (58).

The expression profile of astrocytes, particularly in the Krabbe disease model, reveals an extremely acute phenotype at the HEP, a response more severe than that to an ischemic insult or infection at peak disease time (55). We conclude that in common with other neurodegenerative disorders (58), reactive astrocytes are a prominent feature of Krabbe, NPC1 and SD.

We examined psychosine as a possible inducer of *Casp11* expression in primary cultures of astrocytes isolated from the brains of twi and their wild-type counterparts, and used LPS as a control, a known inducer of its expression. LPS-treated astrocytes of both genotypes robustly induced *Casp11* expression, but exposure to toxic amounts of psychosine did not induce *Casp11*. Of note, in a study by O’Sullivan and Dev, exposure of astrocytes to psychosine caused cell death, but had no effect on the expression of pro-inflammatory cytokines IL6, TNFα, and IL1β. However, psychosine potentiated LPS induction of these cytokines (61). They also found that pre-treatment of these cultures with fingolimod pFTY720, a sphingosine 1-phosphate (S1P) receptor agonist that resembles S1P, attenuated the effects of psychosine and LPS by reducing cell death and cytokine production. Moreover, pFTY720-treated organotypic cerebellar slices resulted in similar findings and inhibition of demyelination. Recently, the same group tested the effects of pFTY20 on twi (85), and described rescue of myelin levels, reduction of reactive microglia and astrocytes concomitant with a modest increase in life span. Given that fingolimod has been shown to have powerful anti-inflammatory effects in different animal models of neurodegeneration (86,87), and that S1P receptors influence expression of cytokines in immune and glial cells (88), it appears likely that inflammasome activation might be mediated, at least in part, by S1P signalling.

To summarize, our investigations identified a profound inflammatory response in Krabbe disease, characterized by a heightened expression and activation of non-canonical and canonical inflammasomes and associated molecules. The reaction starts early in life and intensifies with advancing age and progression of the neuropathological injury. Taken together, the data suggest that this inflammatory response is likely to contribute to the neuropathogenesis of Krabbe disease. Similar findings, however, were identified in NPC1, SD and the Lys toxin model of demyelination, thus pointing to a shared innate immune response that is engaged in these neurodegenerative conditions and is a mechanism likely common to many such diseases.

We hypothesize that activated caspase-11 in microglia/macrophages cleaves and activates Il-1α and Gsdmd, forming pores and allowing the release of Il-1α and other cytokines, which in turn activates astrocytes, cells that abundantly express IL-IR. Although we acknowledge that the understanding of these processes remains limited, and that the conclusions are drawn from: (1) finding an association between inflammasome induction and disease pathology; and (2) prior research in related diseases that demonstrated the pathogenicity of activated inflammasomes in these disorders, defining the precise molecular triggers of inflammasome activation in the diseases studied here are of paramount importance, and further investigations are currently underway to identify the most plausible candidates.

Deciphering inflammatory mechanisms that underpin the pathogenesis of Krabbe disease, and indeed those of other neurodegenerative disorders, is essential to inform the design of future therapeutic stratagems. Our findings provide a tangible route to test whether modulation of the innate immune response, on its own or in combination with established approaches, can address some of the challenges encountered in the treatment of these devastating diseases.

## Materials and Methods

### Mice

Twitcher twi-2J (#000845, C57BL/6 J-*Galc^twi-2J^*) (32,33) and SD knockout (#002914, B6; 129S4-*Hexb^tm1Rlp/J^*) (46) mice were obtained from The Jackson Laboratory (Bar Harbor, ME, USA), and tissue samples from NPC1 (*Npc1^−/−^*, BALB/*cNctr-Npc1^m1N^/J*) (51) animals were a kind gift from Prof Platt (Department of Pharmacology, University of Oxford, UK). All studies were conducted using protocols approved under license by the UK Home Office (Animals Scientific Procedures Act, 1986). *Galc* and *Hexb* genotype was determined by PCR essentially as described elsewhere (89). The approved HEP applied to mice throughout this study was defined as the loss of between 10 and 15% from the maximum achieved weight.

### Lys induction of demyelination

C57Bl6 female mice aged 10–12 weeks were obtained from Charles River (Margate, UK). A demyelination lesion was created by injection of 1% Lysophosphatidylcholine (LPC grade, Sigma-Aldrich, Gillingham, UK) into the ventral white matter of the spinal cord at the level of Th13/L1 through the intervertebral space. Animals were sacrificed 5-days post-lesion.

### Tissue processing

Mice were killed by CO_2_ asphyxiation and organs snap-frozen on dry-ice, or given a lethal dose of pentobarbital and transcardially perfused with ice-cold phosphate-buffered saline (PBS), followed by a solution of 4% paraformaldehyde (pH 7.4) in PBS. Tissue was post-fixed in the same fixative for a few hours, and incubated in 20–30% sucrose overnight at 4°C. The severed spinal cord and sciatic nerves were placed on top of the brain, and 12–15 μm cryosections stored at −80°C.

### Immunohistochemical staining and RNA ISH

IHC staining of non-perfused tissue was performed as previously described (59). Briefly, sections were warmed at room temperature (RT), washed in PBS and fixed in 4% paraformaldehyde (pH 7.4) for 10 min, blocked and permeabilized for 1 h at RT and incubated with primary antibodies at 4°C overnight. Followed by several PBS washes and incubation with secondary antibodies for 1–2 h at RT, washed again and mounted with Prolong Gold medium containing DAPI (4′,6-diamidino-2-phenylindole) (Invitrogen # P36931). Antibody details are given in Supplementary Material, Table S1.

For combined mRNA ISH and IHC staining, twi, Sandhoff and Lys-treated mice were perfused and NPC1 mice killed by CO_2_ asphyxiation. The ISH RNAscope Multiplex FL v2 procedure was performed first, following Advanced Cell Diagnostics/BioTechne’s recommendations for either fixed-frozen or fresh-frozen sections. Mouse-specific RNAscope mRNA probes were from Advanced Cell Diagnostics/BioTechne (ACD): Mm-Caspase1 (#404551), Mm-Caspase4 (which is mouse-specific caspase11) (# 589511) and Mm-Gsdmd (#537601). Signal was developed with dye Opal™ 570 (Akoya Biosciences, #FP1488001KT) diluted 1/1500. Following the ISH protocol, sections were processed for IHC. Confocal microscopy was performed using a Leica Sp5 ultra-high-speed inverted confocal microscope. About 25–29 *z*-stack confocal images were taken and analysed with Fiji software.

### RT-qPCR

Tissue RNA extraction, first-strand cDNA synthesis and relative quantitation by Real-time quantitative PCR of total RNA were performed as described elsewhere (59). About 1 μL of reverse transcription reaction was mixed with 300 nmol of each primer (Supplementary Material, Table S2) and Power SYBR green PCR master mix (Applied Biosystems #4367659) to a final volume of 20 μL. RT-qPCR was performed on three different animals per group (matched for age and sex) in triplicate, with Gapdh as the internal control. The analysis was calculated with the Delta–delta Ct method, and graphically represented as mean ± STDEV (standard deviation). Significance was analysed with unpaired Student’s *t*-test against wild-type mice, and a *P*-value ≤ 0.05 (^*^) was considered statistically significant.

### Western blotting

Tissue protein extraction, polyacrylamide-gel electrophoresis and immunoblotting were performed as described before (59). In brief, 5–250 μg of protein extracts were heated at 90°C and run in 4–15% linear gradient gels (161–1122; Bio-Rad) or in 8–15% gels, and transferred onto polyvinylidene difluoride membranes (Millipore #IPV00010). Blots were processed with primary and horseradish peroxidase-conjugated secondary antibodies (Supplementary Material, Table S1) and developed with Amersham ECL™ western blotting Analysis System (GE Healthcare #RPN2109). Blots were stripped and re-probed with other antibodies following Abcam’s recommendations.

### Purification and labelling of myelin debris

Twi mice aged 36 days were killed by CO_2_ asphyxiation and brain myelin extracted and labelled as described (90). Myelin pellets were resuspended in sterile PBS and their concentration measured using a Pierce Thermo scientific kit (#23227) and stored at −80°C in 100 mg/ml aliquots.

### Isolation, treatment and staining of primary astrocyte cultures

New-born (on the day of birth and up to 4 days post-birth) mouse pups were killed and astrocytes purified from cerebral cortices following the method as described (91). Cells were plated on flasks coated with poly-D-lysine (Sigma # P4707) and incubated at 37°C in a 5% CO_2_ incubator. The medium was changed 2 days post-plating and every 3 days thereafter.

To obtain cultures enriched in astrocytes, flasks were shaken at 180 rpm for 30 min on an orbital shaker for microglia removal once confluent (~7–8 days). Medium was replaced and flasks shaken at 240 rpm for 6 h to remove oligodendrocytes. Astrocytes were harvested and re-plated, and after 12–14 days split at a density of 3–5 × 10^4^ cells/well into 4-well Lab-Tek chamber slides. About 24–48 h later, individual astrocyte wells were cultured in the presence of one of the following compounds for about 18 h: 100 ng/ml LPS (Sigma # L2654-1MG), 5 μg/ml myelin debris (isolated, purified and labelled as aforementioned), 10 μM galactosylsphingosine (Sigma # P9256-1MG) and 10 μM β-galactosylceramide (Sigma # C4905), or culture medium without compounds (untreated). Cultured astrocytes were washed in PBS, fixed in 4% paraformaldehyde (pH 7.4) for 30 min at RT and processed for ISH with RNAscope probe Caspase-11 and IHC for Gfap following ACD’s recommendations for the treatment of adherent cells cultured on coverslips.

### Statistical analysis

Experimental groups were sex- and age-matched. Each data point in the graphs and each lane in western blots represents a different animal. Statistical tests were performed using GraphPad Software (GraphPad Prism v5.0). All results are presented as the mean ± STDEV. Statistical comparisons were made using one-way ANOVA with Bonferroni’s multiple post hoc test and the Student’s *t*-test when comparing two samples. Values with *P* ≤ 0.05 were considered significant. ^*^P ≤ 0.05; ^*^^*^P ≤ 0.01; ^*^^*^^*^P ≤ 0.001 and ^*^^*^^*^^*^P ≤ 0.0001.

## Supplementary Material

Supplementary_Material_Fig_S1_HMG_(02122022)_300_tiff_ddac299

Supplementary_Material_Fig_S2_HMG_(02122022)_300_tiff_ddac299

Supplementary_Material_Fig_S3_HMG_(02122022)_300_tiff_ddac299

Supplementary_Material_Fig_S4_HMG_(02122022)_300_tiff_ddac299

Supplementary_Material_Fig_S5_HMG_(02122022)_300_tiff_ddac299

Supplementary_Material_Fig_S6_HMG_(02122022)_300_tiff_ddac299

Supplementary_Material_Fig_S7_HMG_(02122022)_300_tiff_ddac299

Supplementary_Material_Fig_S8_HMG_(18112022)_300_tiff_ddac299

Supplementary_Material_Table_S1_(02122022)_ddac299

Supplementary_Material_Table_S2_(02122022)_ddac299

LEGENDS_TO_SUPPLEMENTARY_MATERIAL_FIGURES_ddac299

## Data Availability

Not applicable.
