# Supplementary figures and images for: Upregulation of non-canonical and canonical inflammasome genes associates with pathological features in Krabbe disease and related disorders
Source: Hum Mol Genet. 2022 Dec 15;32(8):1361–79. doi: 10.1093/hmg/ddac299 (PMC10077509; doi:10.1093/hmg/ddac299)

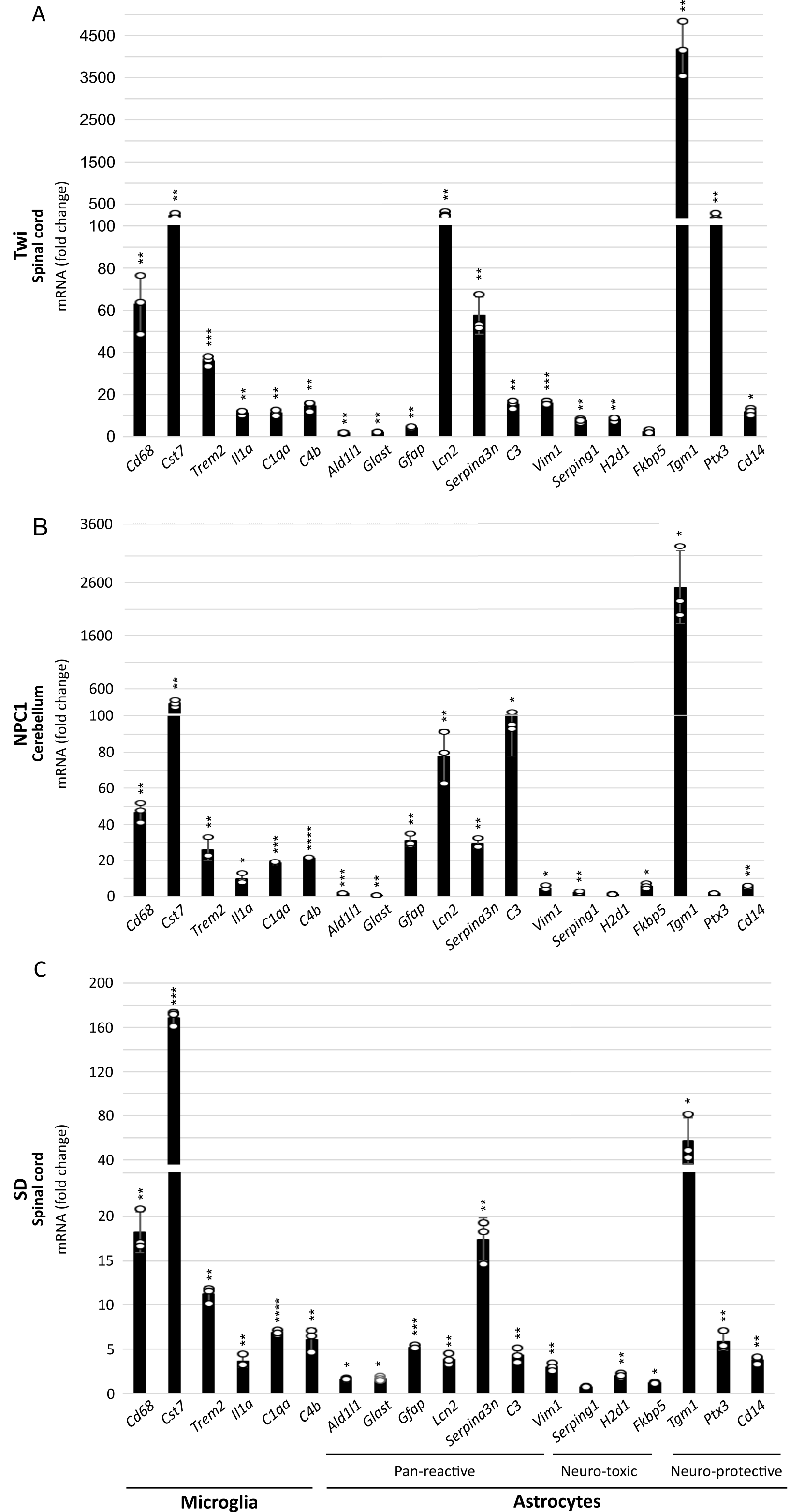

Supplement: Supplementary_Material_Fig_S1_HMG_(02122022)_300_tiff_ddac299 [file supplementary_material_fig_s1_hmg_(02122022)_300_tiff_ddac299.zip › Supplementary_Material_Fig_S1_HMG_(02122022)_300_tiff_ddac299.tif]

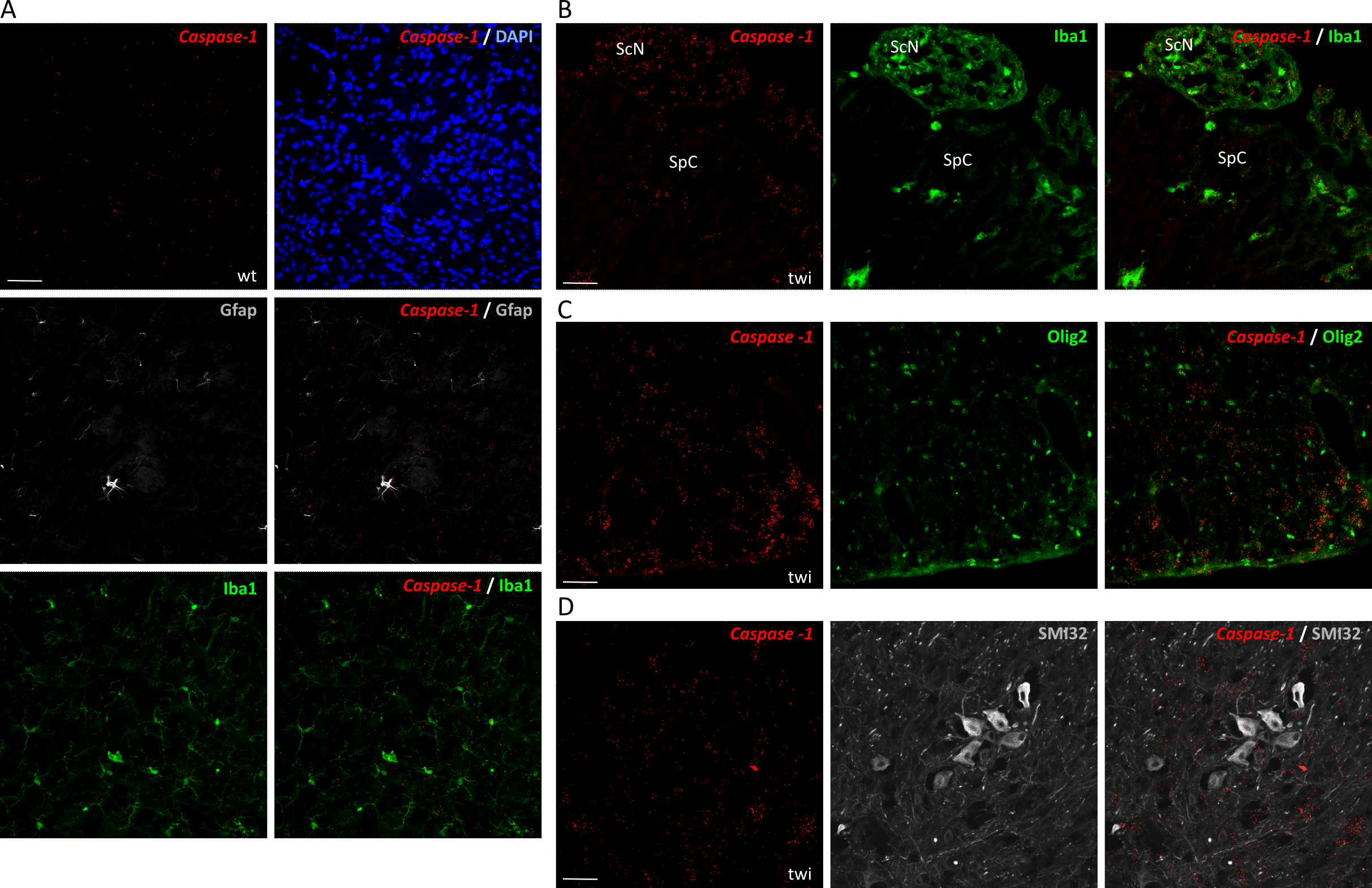

Supplement: Supplementary_Material_Fig_S2_HMG_(02122022)_300_tiff_ddac299 [file supplementary_material_fig_s2_hmg_(02122022)_300_tiff_ddac299.zip › Supplementary_Material_Fig_S2_HMG_(02122022)_300_tiff_ddac299.tif]

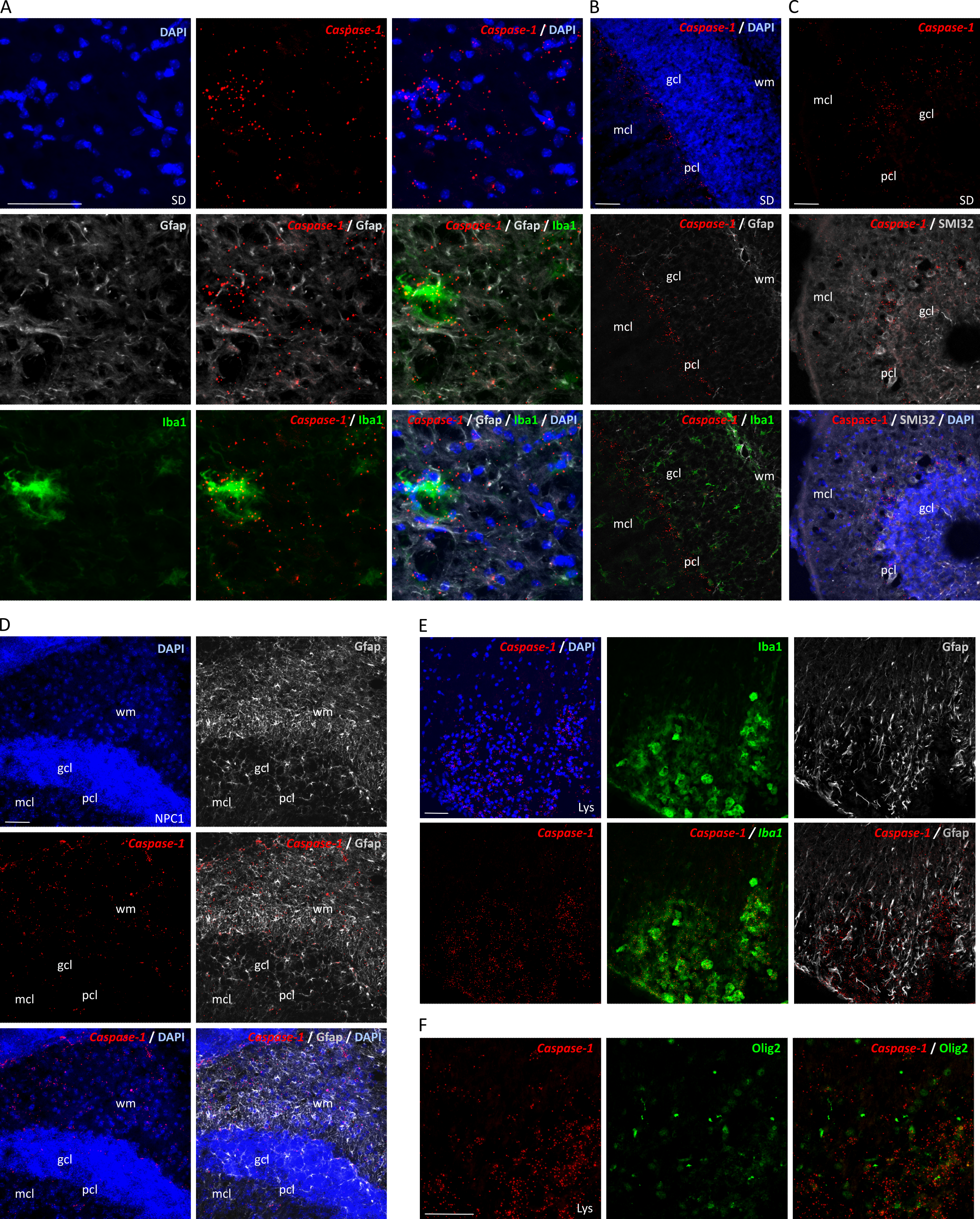

Supplement: Supplementary_Material_Fig_S3_HMG_(02122022)_300_tiff_ddac299 [file supplementary_material_fig_s3_hmg_(02122022)_300_tiff_ddac299.zip › Supplementary_Material_Fig_S3_HMG_(02122022)_300_tiff_ddac299.tif]

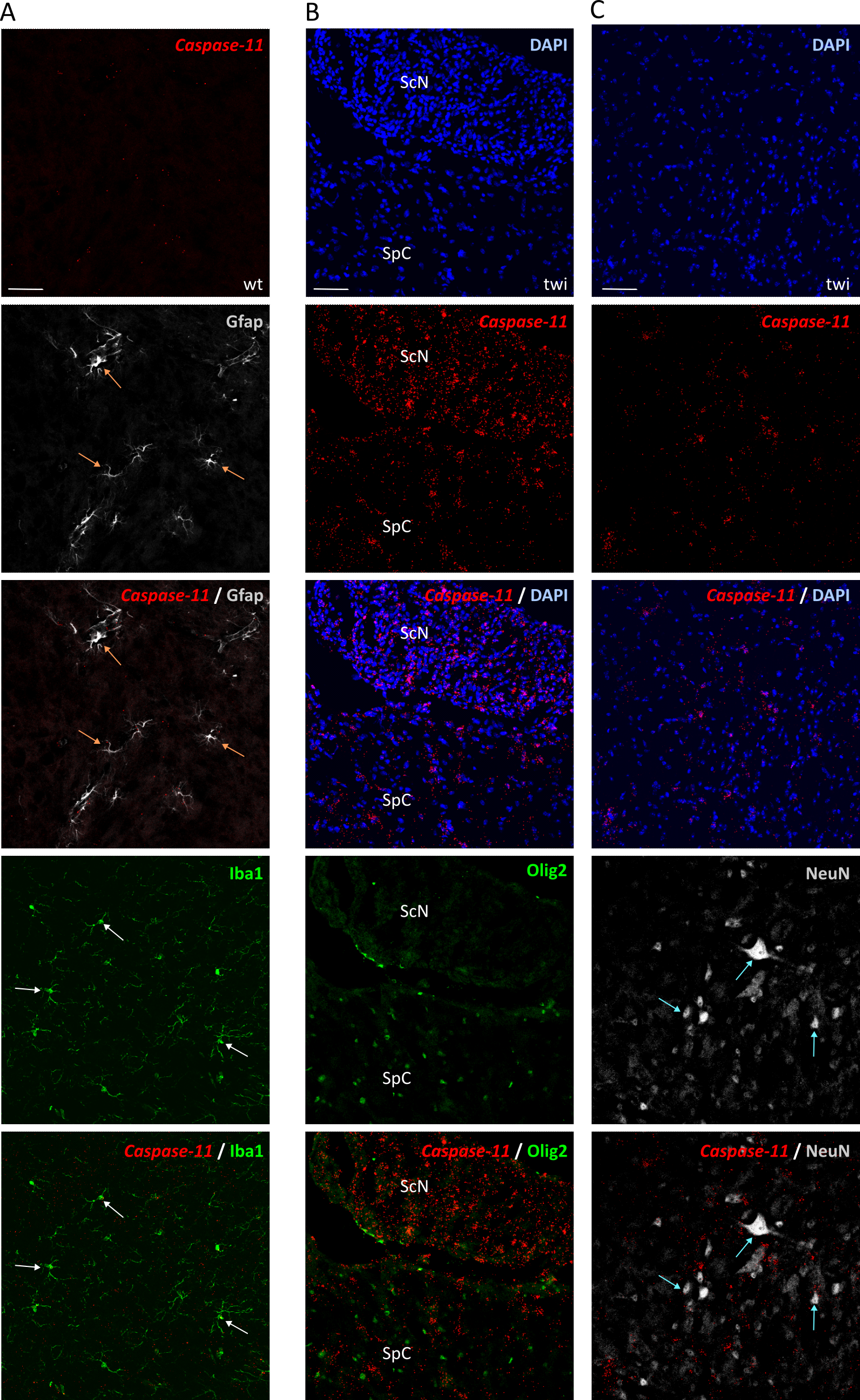

Supplement: Supplementary_Material_Fig_S4_HMG_(02122022)_300_tiff_ddac299 [file supplementary_material_fig_s4_hmg_(02122022)_300_tiff_ddac299.zip › Supplementary_Material_Fig_S4_HMG_(02122022)_300_tiff_ddac299.tif]

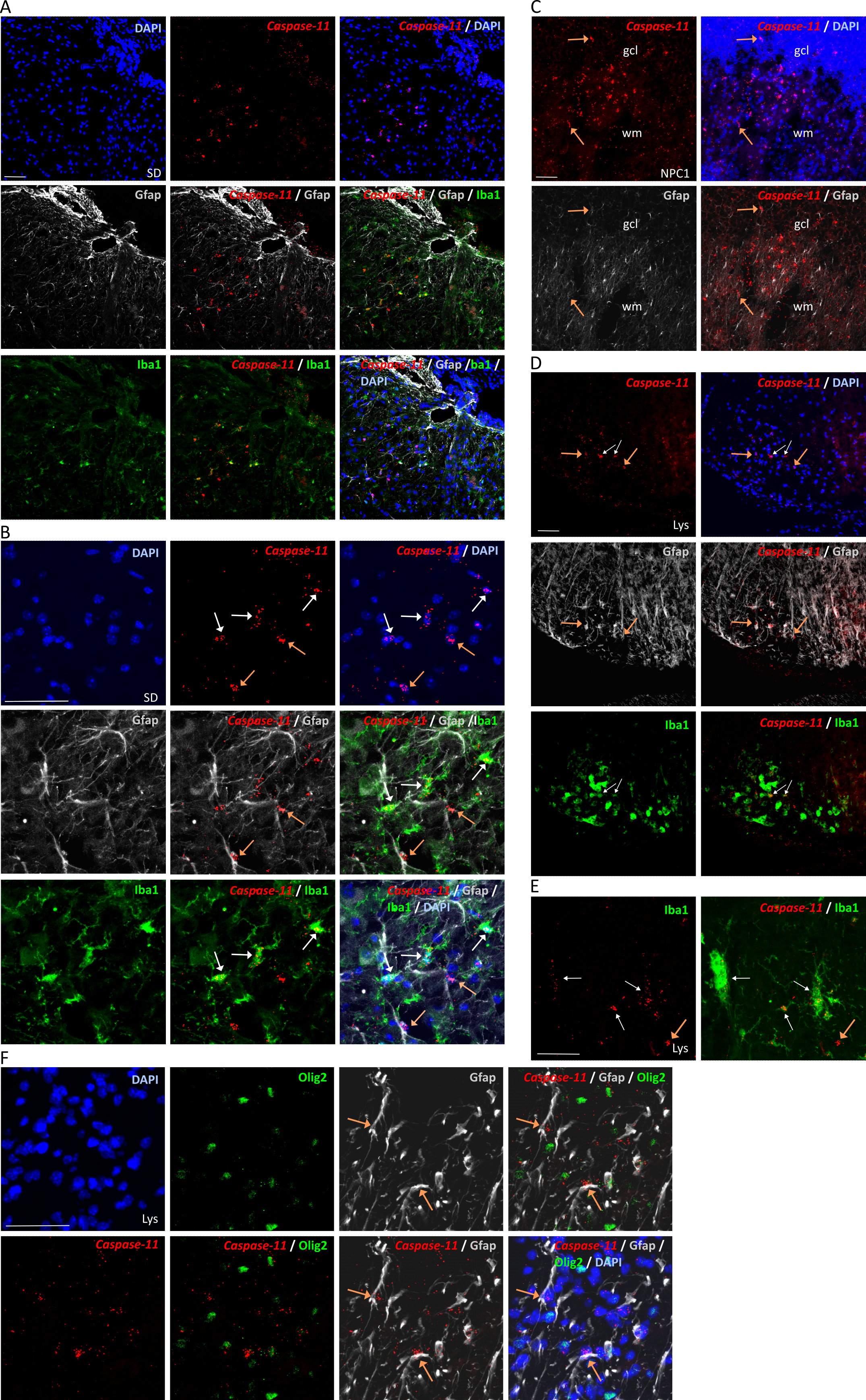

Supplement: Supplementary_Material_Fig_S5_HMG_(02122022)_300_tiff_ddac299 [file supplementary_material_fig_s5_hmg_(02122022)_300_tiff_ddac299.zip › Supplementary_Material_Fig_S5_HMG_(02122022)_300_tiff_ddac299.tif]

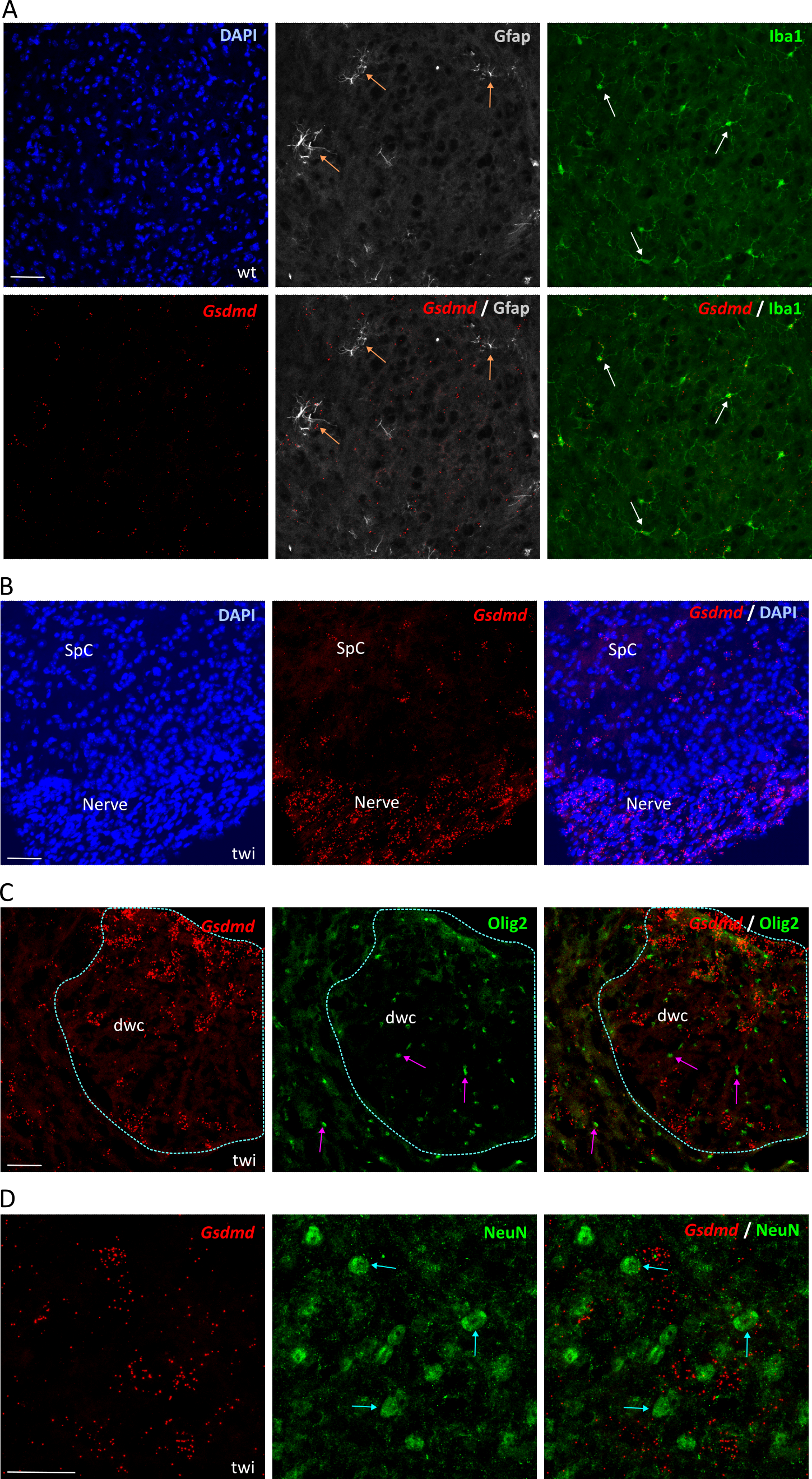

Supplement: Supplementary_Material_Fig_S6_HMG_(02122022)_300_tiff_ddac299 [file supplementary_material_fig_s6_hmg_(02122022)_300_tiff_ddac299.zip › Supplementary_Material_Fig_S6_HMG_(02122022)_300_tiff_ddac299.tif]

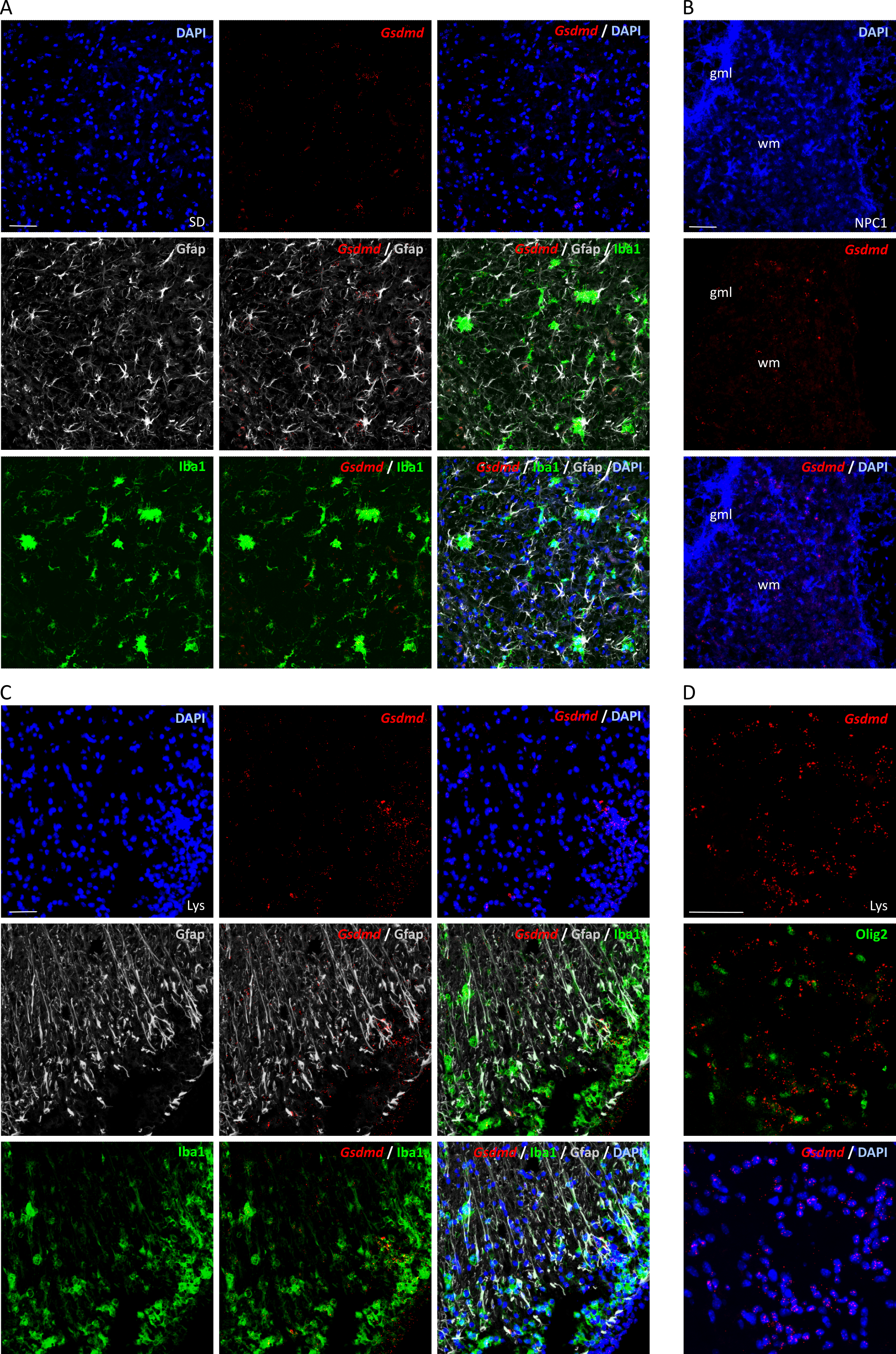

Supplement: Supplementary_Material_Fig_S7_HMG_(02122022)_300_tiff_ddac299 [file supplementary_material_fig_s7_hmg_(02122022)_300_tiff_ddac299.zip › Supplementary_Material_Fig_S7_HMG_(02122022)_300_tiff_ddac299.tif]

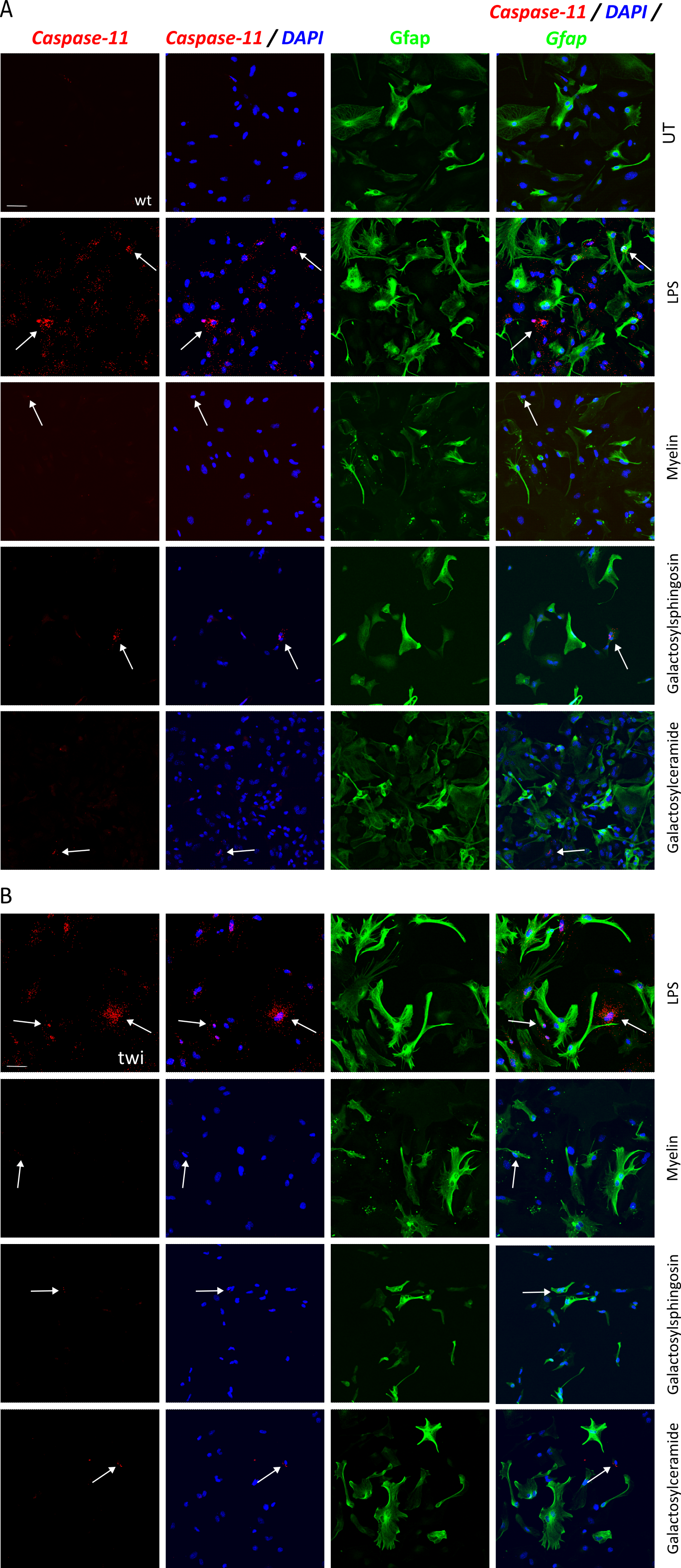

Supplement: Supplementary_Material_Fig_S8_HMG_(18112022)_300_tiff_ddac299 [file supplementary_material_fig_s8_hmg_(18112022)_300_tiff_ddac299.zip › Supplementary_Material_Fig_S8_HMG_(18112022)_300_tiff_ddac299.tif]
