# Supplementary material for: Upregulation of non-canonical and canonical inflammasome genes associates with pathological features in Krabbe disease and related disorders
Source: Hum Mol Genet. 2022 Dec 15;32(8):1361–79. doi: 10.1093/hmg/ddac299 (PMC10077509; doi:10.1093/hmg/ddac299)
Supplement: LEGENDS_TO_SUPPLEMENTARY_MATERIAL_FIGURES_ddac299 [file legends_to_supplementary_material_figures_ddac299.docx]

**LEGENDS TO SUPPLEMENTARY MATERIAL FIGURES**

**Supplementary Material Figure S1.** The severity of phenotype in reactive glia generally correlates with the degree of inflammasome gene expression in murine models of lysosomal storage diseases. Gene expression was examined by RT-qPCR at the humane end point (HEP) and performed in wild type and mutant animals (n=3 distinct animals per group) in triplicate: (A) twitcher (Twi), spinal cord; (B) Niemann-Pick Type C1 disease (NPC1), cerebellum; and (C) Sandhoff disease (SD), spinal cord. *Cd68* (Cluster of differentiation 68), *Cst7* (Cystatin F), *Trem2* (Triggering receptor expressed on myeloid cells 2), *Il1a* (Interleukin 1 alpha), *C1qa* (Complement component 1qa), *C4b* (Complement component 4b), *Adl1l1* (Aldehyde dehydrogenase 1 family member L1), *Glast* (Glutamate aspartate transporter), *Lcn2* (Lipocalin 2), *Serpina3n* (Alpha-1-antichymotrypsin), *C3* (Complement component 3), *Vim1* (Vimentin), *Serping1* (Plasminogen activator inhibitor-1), *H2d1* (Histocompatibility 2 D region locus 1), *Fkbp5* (FKBP prolyl isomerase 5), *Tgm1* (Transglutaminase 1), *Ptx3* (Pentraxin 3), and *Cd14* (Cluster of differentiation 14). Data is expressed relative to wild type animals as mean±STDEV and tested by unpaired Student’s t-test. Symbols above error bars are mutant values versus wild type. The absence of a symbol indicates the test did not reach significance. ∗P ≤ 0.05; ∗∗P ≤ 0.01; ∗∗∗P ≤ 0.001 and ∗∗∗∗P ≤ 0.0001.

**Supplementary Material Figure S2.** Expression of caspase-1 is low in wild type mice and does not co-localise with Oligo2 or SMI32 stained cells in twitcher. Representative sections of spinal cord from wild-type (wt) (A), and twitcher (twi) (B-D) at the HEP, co-stained by *in situ* hybridization for caspase-1 mRNA and immunohistochemically for astrocytes with Gfap (Glial fibrillary acidic protein), microglia/macrophages with Iba1 (Ionized calcium binding adaptor molecule 1), oligodendroglia with Olig2 (Oligodendrocyte transcription factor 2) and neurones with SMI32 (non-phosphorylated neurofilament protein). Nuclear stain is DAPI (4′,6-diamidino-2-phenylindole). Scale bars: 50μm.

**Supplementary Material Figure S3.** Canonical caspase-1 localises principally to reactive microglia/macrophage in Sandhoff disease (SD), Niemann–Pick type C1 disease (NPC1), and the lysolecithin (Lys) toxin model of demyelination in mice. Representative sections of brain stem (A) and cerebellum (B, C) from SD, and cerebellum from NPC1 (D) mice at their HEP, or spinal cord from wild-type mice injected with lysolecithin and killed five days post-injection (E, F). Co-staining was performed by *in situ* hybridization for caspase-1 mRNA and immunohistochemically for astrocytes with Gfap (Glial fibrillary acidic protein), microglia/macrophages with Iba1 (Ionized calcium binding adaptor molecule 1), neurones with non-phosphorylated neurofilament protein (SMI32), oligodendroglia with Olig2 (Oligodendrocyte transcription factor 2). Nuclear stain is DAPI (4′,6-diamidino-2-phenylindole). Cerebellar granular cell layer (gcl), molecular cell layer (mcl), Purkinje cell layer (pcl), and white matter (wm). Scale bars: 50μm.

**Supplementary Material Figure S4.** Expression of caspase-11 is low in wild-type mice and does not co-localise with Oligo2 or NeuN stained cells in twitcher. Representative sections of spinal cord from wild-type (wt) (A), and twitcher (twi) (B, C) at the HEP, co-stained by *in situ* hybridization with caspase-11 mRNA and immunohistochemically for astrocytes with Gfap (Glial fibrillary acidic protein), microglia/macrophages with Iba1 (Ionized calcium binding adaptor molecule 1), oligodendroglia with Olig2 (Oligodendrocyte transcription factor 2), and neurones with NeuN (Neuronal Nuclei). White, fuchsia, orange and blue arrows point to microglia/macrophages, astrocytes, oligodendroglia, and neurones, respectively. Nuclear stain is DAPI (4′,6-diamidino-2-phenylindole). Note the intense staining for caspase-11 RNA in the sciatic nerve (ScN) compared with spinal cord (SpC) in B. Scale bars: 50μm.

**Supplementary Material Figure S5.** Non-canonical caspase-11 localises to both reactive microglia/macrophages and astrocytes in Sandhoff disease (SD), Niemann–Pick type C1 disease (NPC1) and the lysolecithin (Lys) toxin model of demyelination. Representative sections of brain stem from SD (A, B), and cerebellum from NPC1 (C) mice at their HEP, and spinal cord from wild-type mice injected with lysolecithin and killed five days post-injection (D-F). Co-staining was performed by *in situ* hybridization with caspase-11 mRNA and immunohistochemically for astrocytes with Gfap (Glial fibrillary acidic protein), microglia/macrophages with Iba1 (Ionized calcium binding adaptor molecule 1), and oligodendroglia with Olig2 (Oligodendrocyte transcription factor 2). Nuclear stain is DAPI (4′,6-diamidino-2-phenylindole). Cerebellar granular cell layer (gcl) and white matter (wm). White and orange arrows point to microglia/macrophages and astrocytes respectively. Note the different cellular distribution of *Casp-11* signal between microglia/macrophages and astrocytes. Scale bars: 50μm.

**Supplementary Material Figure S6.** Expression of *Gsdmd* is low in wild type mice and does not co-localise with Oligo2 or NeuN stained cells in twitcher. Representative sections of spinal cord from wild-type (wt) (A), and twitcher (twi) (B-D) at the HEP, co-stained by *in situ* hybridization for *Gsdmd* mRNA and immunohistochemically for astrocytes with Gfap (Glial fibrillary acidic protein), microglia/macrophages with Iba1 (Ionized calcium binding adaptor molecule 1), oligodendroglia with Olig2 (Oligodendrocyte transcription factor 2), and neurones with NeuN (Neuronal Nuclei). Note the strong *Gsdmd* staining in spinal nerve (B) and dorsal white column (dwc) of spinal cord (C) outlined with blue dashes in mutant mice. White, fuchsia, orange and blue arrows point to microglia/macrophages, astrocytes, oligodendroglia, and neurones, respectively. Nuclear stain is DAPI (4′,6-diamidino-2-phenylindole). Scale bars: 50μm.

**Supplementary Material Figure S7.** *Gsdmd* localises principally to reactive microglia/macrophage in Sandhoff disease (SD), Niemann–Pick type C1 disease (NPC1) and the lysolecithin (Lys) toxin model of demyelination. Representative sections of brain stem from (SD) (A), cerebellum from NPC1 (B) mice at their HEP, or spinal cord from wild-type mice injected with lysolecithin and killed five days post-injection (C, D). Co-staining was performed by *in situ* hybridization with *Gsdmd* and immunohistochemically for astrocytes with Gfap (Glial fibrillary acidic protein), microglia/macrophages with Iba1 (Ionized calcium binding adaptor molecule 1), and oligodendroglia with Olig2 (Oligodendrocyte transcription factor 2). Nuclear stain is DAPI (4′,6-diamidino-2-phenylindole). Cerebellar granular cell layer (gcl) and white matter (wm). Scale bars: 50μm.

**Supplementary Material Figure S8.** Myelin debris, galactosylsphingosine or galactosylceramide do not prime caspase-11 in primary astrocyte cultures isolated from twitcher. Representative sections of cultured primary astrocytes isolated from-wild type (wt) (A) or twitcher (twi) (B) brains, untreated (UT) or treated with LPS, myelin debris, galactosylsphingosine (psychosine), and galactosylceramide. Co-staining was performed by *in situ* hybridization with caspase-11 mRNA and immunohistochemically for astrocytes with Gfap (Glial fibrillary acidic protein). Nuclear stain is DAPI (4′,6-diamidino-2-phenylindole). Arrows point to caspase-11 positive cells. Scale bars: 50μm.
